# From Molecules to Bioaggregates: Unraveling the Photoexcitation Dynamics of Intracellularly Self‐Assembled Thiophene‐Based Fibers

**DOI:** 10.1002/smsc.202500241

**Published:** 2025-07-28

**Authors:** Filippo Monti, Ludovico Aloisio, Nicol Spallacci, Mattia Zangoli, Antonella Treglia, Ariel Garcìa Fleitas, Michele Guizzardi, Soraia Flammini, Matteo Moschetta, Giuseppe Maria Paternò, Francesca Di Maria, Guglielmo Lanzani

**Affiliations:** ^1^ Institute for Organic Synthesis and Photoreactivity (ISOF) National Research Council of Italy (CNR) Via P. Gobetti 101 I‐40129 Bologna Italy; ^2^ Dipartimento di Fisica Politecnico di Milano Piazza L. da Vinci 32 20133 Milano Italy; ^3^ Center for Nano Science and Technology Istituto Italiano di Tecnologia Via Rubattino 81 20134 Milano Italy

**Keywords:** biofibers, density functional theory calculations, intracellular self‐assembly, oligothiophene, photoexcitation dynamics

## Abstract

The spontaneous self‐assembly of supramolecular structures within biological environments offers a powerful strategy for developing functional biomaterials capable of interacting with living systems. By integrating optical spectroscopy with quantum‐chemical calculations, it is demonstrated that the assembly of 2,6‐diphenyl‐3,5‐dimethyl‐dithieno[3,2‐b:2′,3′‐d]thiophene‐4,4‐dioxide (DTTO) molecules into fibers within cells leads to distinct photophysical properties and enhanced biocompatibility. Photoluminescence and transient absorption spectroscopy reveal weak intermolecular interactions in the fibers, which are sufficient for supporting energy and charge transport. Additionally, the observation of stimulated emission suggests that optical gain can be achieved within these fibers. Biological assays on *Escherichia coli* exposed to DTTO provide insight into the material's stability and biocompatibility. While DTTO aggregates formed in aqueous environments exhibit phototoxicity, DTTO fibers produced by cells do not. Time‐resolved spectroscopy suggests that this difference arises from the absence of long‐lived photoexcited states in the fibers, a consequence of their distinct molecular packing. These findings underscore the fundamental role of cell‐guided self‐assembly in tuning optical properties and, consequently, in modulating biological interactions, positioning DTTO fibers as promising candidates for biocompatible electronic interfaces and intracellular applications.

## Graphical abstract

Cell‐guided self‐assembly of thiophene based molecules inside living cells produces photostable, biocompatible nanofibers exhibiting J‐like excitonic coupling and stimulated emission. Integrated spectroscopic and quantum‐chemical analyses reveal unique photophysical behavior and low phototoxicity, positioning these supramolecular structures as promising intracellular optoelectronic components.

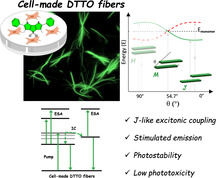

## 1 Introduction

Self‐assembly within living cells is a ubiquitous phenomenon characterized by the spontaneous organization of biomolecular building blocks into functional structures essential for cellular processes.^[^ ^1^, ^2^ ^]^ Inspired by these natural mechanisms, the intracellular assembly of synthetic components has emerged as a promising strategy for generating artificial nanostructures with biofunctionalities that target specific purposes.^[^ ^3^, ^4^ ^]^ Although this approach has been investigated in a limited number of studies, findings consistently indicate that synthetic building blocks—introduced as preformed units or generated in situ through self‐driven chemical reactions—encode structural information within their covalent framework, guiding their organization into well‐defined supramolecular structures even in complex biological environments.^[^ ^5^, ^6^, ^7^, ^8^, ^9^, ^10^, ^11^, ^12^ ^]^

The potential of this approach lies in its ability to overcome natural barriers, enabling: 1) in situ constitution of functional nanostructures, allowing for precise therapeutic or diagnostic interventions directly within specific biological targets; 2) the intracellular production of biomaterials or therapeutic agents with high specificity and efficiency; and 3) a deeper understanding of cellular dynamics at the nanoscale, offering insights into fundamental biological processes, such as signal transduction, molecular trafficking, and cellular responses.^[^ ^13^, ^14^, ^15^, ^16^, ^17^ ^]^ Importantly, such molecular‐level integration and control are particularly attractive for applications in bioelectronics, where organic functional materials can bridge electronic systems and living tissues, enabling soft, biocompatible, and responsive interfaces.^[^ ^18^, ^19^, ^20^, ^21^ ^]^

Here, we investigate the photophysics of crystalline, fluorescent, and conductive fibers formed by the intracellular self‐assembly of a thiophene‐based small molecule, namely 2,6‐diphenyl‐3,5‐dimethyl‐dithieno[3,2‐b:2′,3′‐d]thiophene‐4,4‐dioxide, DTTO.^[^ ^22^, ^23^, ^24^ ^]^ These fibers display nanometric cross‐sections (10–100 nm in height and 100–300 nm in width), with lengths extending from hundreds of nanometers to several micrometers.

The biogenesis of DTTO fluorescent fibers in living cells was first reported in 2011.^[^ ^22^ ^]^ Since then, extensive experimental investigations have demonstrated that: 1) the aggregation phenomenon is universal and independent of the cell type; 2) the cellular metabolic activity provides unique conditions, such as molecular crowding, enzymatic activity, or localized environmental factors, for guiding fiber growth; and 3) in their aggregated form, DTTO fibers exhibit charge conductivity.^[^ ^25^, ^26^, ^27^, ^28^ ^]^ It has been hypothesized that DTTO fibers within cells grow through a nonclassical nucleation pathway involving the formation of liquid‐like droplets during nucleation.^[^ ^29^, ^30^, ^31^ ^]^ These DTTO droplets accumulate, coalesce, and ultimately lead to the formation of unique crystalline fibers consisting solely of DTTO molecules with a characteristic molecular packing.^[^ ^24^ ^]^ Proteins, while not integrated into the fiber crystalline structure, can be present as a surface coating, perhaps functioning as a functional scaffold.

Although cell‐made DTTO fibers have been investigated for over a decade, their electronic structure and photoexcited‐state dynamics remain largely unexplored. This study aims to fill that gap by comparing the electronic properties of DTTO in its monomeric form (i.e., in DMSO solution) with those in its aggregated state, specifically as fibers. We present a comprehensive analysis, combining steady‐state and time‐resolved absorption and emission data, spanning femtoseconds to nanoseconds, for both sample types.

By systematically analyzing key parameters—such as excited‐state lifetimes, absorption cross‐sections, and deactivation pathways—we gain critical insights into the excitonic coupling, energy transfer, and electronic relaxation mechanisms that govern the optoelectronic behavior of DTTO fibers. In addition, density functional theory (DFT) calculations on dimer models complement this study by identifying potential aggregation motifs and assessing their influence on the optical and electronic properties of DTTO assemblies. Time‐resolved spectroscopy further correlates the high stability/biocompatibility of DTTO fibers with the absence of long‐lived photoexcited states, highlighting the pivotal role of their distinct molecular packing in facilitating rapid excited‐state deactivation. Our findings suggest that intracellular DTTO assembly not only enhances the photostability of the material but also minimizes the risk of phototoxic effects, as demonstrated in biological assays on *Escherichia coli*, making DTTO fibers a promising and safe platform for biooptoelectronic applications in living systems.

## 2 Results and Discussion

### 2.1 Fiber Formation, Absorbance, and Emission

**Figure 1:**
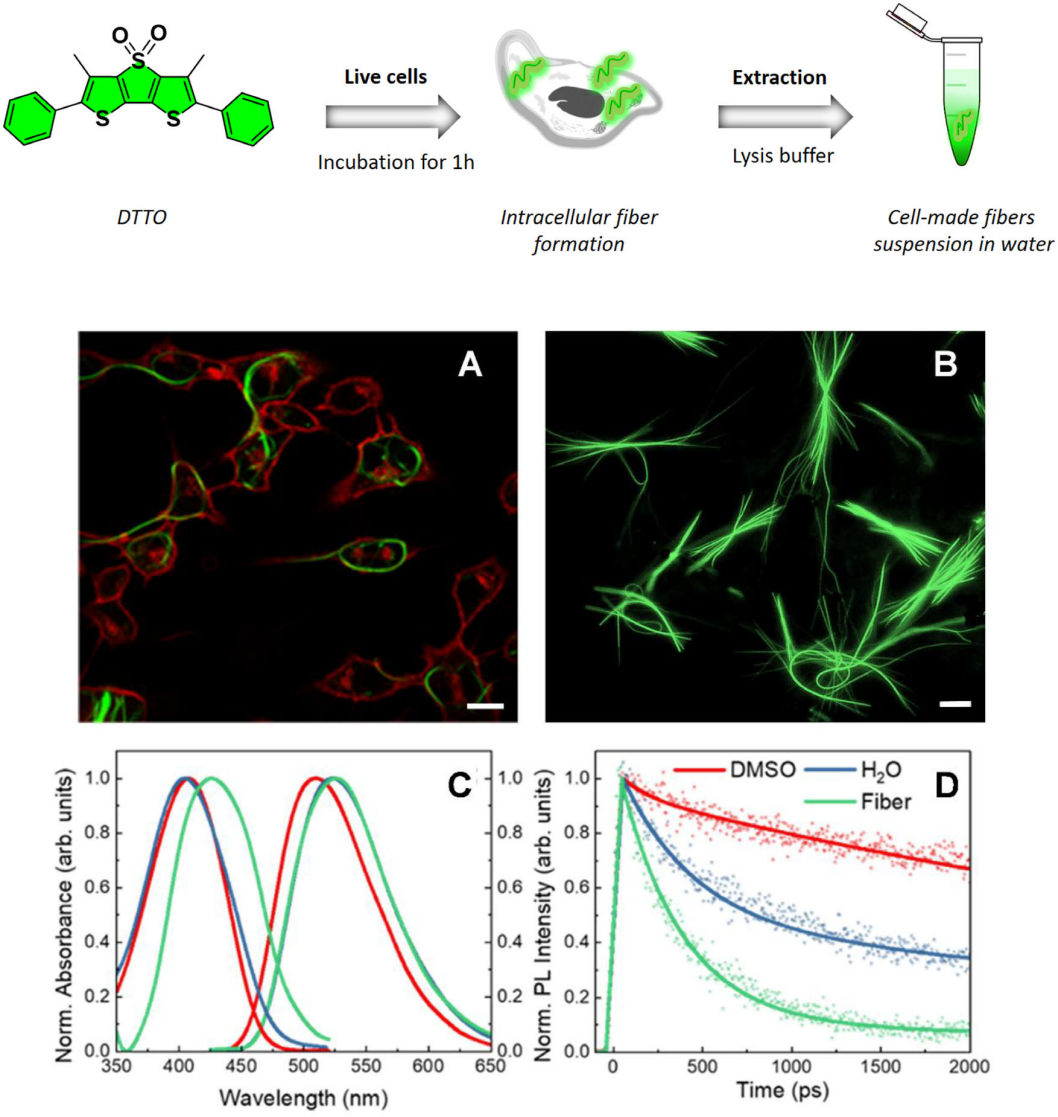
(Top) Sketch of the preparation of DTTO fibers within cells. A) Single plane of laser scanning confocal microscopy images of C2C12 cells stained with DTTO, after fiber formation, taken 24 h after treatment. Cells were incubated with CellMask Deep Red plasma membrane stain for 5 min before imaging to highlight the cell membranes (red). Scale bar: 10 μm. B) Epifluorescent optical micrographs of DTTO fibers after isolation, deposited on a glass substrate. Scale bar: 10 μm. C) Absorbance and PL spectra of DTTO dissolved in DMSO (red), aggregates after precipitation in water (blue), and cell‐made fibers suspended in water (green). The absorption spectrum of fibers is corrected for scattering. D) PL decays of DTTO in DMSO (red), water (blue), and fibers (green). Double exponential fits are represented by continuous lines and experimental data by dots.

**Figure** **1**A shows an example of DTTO fibers self‐assembled inside C2C12 cells. These fibers are formed through the spontaneous aggregation of DTTO molecules in the intracellular environment, driven by the unique physicochemical conditions of the cytoplasm.^[^ ^22^, ^24^ ^]^ Fibers were then extracted from cells using a lysis buffer, following the method previously reported by us, to avoid structural or chemical modifications during the purification process (see sketch in Figure 1). Figure 1B reports a fluorescence optical microscopy image of isolated fibers deposited on a glass substrate. Optical properties were studied in aqueous suspension for fibers and in DMSO solution for monomers. Notably, DTTO molecules in water quickly and spontaneously self‐assemble into crystalline aggregates, which differ significantly from the fiber (Figure S1, Supporting Information).^[^ ^24^ ^]^ For the sake of completeness, we also investigated these samples as a further reference to unravel the optoelectronic properties of DTTO in its aggregated states.

Figure 1C reports the scattering‐corrected and normalized absorption and emission spectra of fibers in comparison with DTTO monomer in DMSO and DTTO aggregate in water. In all the spectra, inhomogeneous broadening masks the vibronic features, resulting in a similar width. The DTTO monomer dissolved in DMSO displays a featureless broad absorption band peaking at 408 nm. The aggregates in water, formed by precipitation upon adding water to a solution of DTTO in DMSO (25 μg mL^−1^ in 1% DMSO v/v), show a similar spectrum but with a slightly blue shift, suggesting the presence of H‐aggregates. In contrast, the suspension of DTTO fibers extracted from cells in water exhibits a clear redshift of the absorption peak, ≈20 nm with respect to that of the monomer. The fiber suspension in water is highly scattering. To disentangle the contributions of scattering and absorption, we applied simple algorithms that leverage the principles of Rayleigh and Mie scattering.^[^ ^32^, ^33^ ^]^ The scattering component was fitted by Equation S1, Supporting Information, and then subtracted (Figure S2, Supporting Information). Fitting revealed a much higher contribution of the Mie domain with respect to Rayleigh's one (see Supporting Information for fitting parameters), consistent with the fiber widths typically ranging from 100 to 300 nm. In all conditions, DTTO exhibits a significant Stokes shift between absorption and emission (PL), in the order of 0.6 eV for the monomer in solution and fibers, and even larger (0.7 eV) for aggregates in water (Figure 1C). The emission spectra are again similar in shape, but the aggregates in water and fibers are redshifted with respect to monomer emission.

Figure 1D shows the PL decay dynamics. In DMSO solution, the DTTO molecule exhibits a nearly single‐exponential decay kinetics, with a lifetime of *τ* = 5.7 ns (≈6% of the amplitude decays with a time constant of 150 ps). The measured PL quantum yield (PLQY) is about 0.85–0.95.^[^ ^22^, ^34^ ^]^ The suspension of DTTO aggregates in water is weakly emitting, with PLQY below 0.1 but without relevant changes in the spectrum, apart from a slight redshift. About half (A_2_ = 0.48) of the emitted light decays with the same kinetics as DTTO in DMSO. The remaining fraction has a lifetime of 350 ± 50 ps. Similarly, the PL spectrum of DTTO fibers closely resembles that of the aggregates in water, however, the PL lifetime is strongly reduced, displaying a predominant fast exponential decay *τ*_1_ = 350 ps (A_1_ = 0.9) followed by a small tail with a time constant *τ*_2_ = 5.7 ns (A_2_ = 0.1). The PLQY is 0.3, and the calculated *τ*_RAD_ is (2.7 ± 0.7) ns.

We assign the emission with *τ* = 5.7 ns to the intrinsic emission of the DTTO molecules, while the faster decay components are attributed to intermolecular interactions within the aggregates, which introduce nonradiative decay pathways that differ between aggregates in water and fibers.

### 2.2 Theoretical Study on DTTO Single Molecule

**Figure 2:**
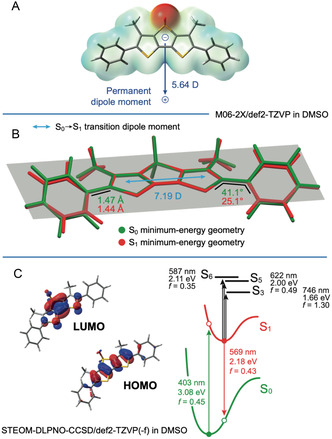
A) Permanent dipole moment of DTTO in DMSO (*C*_s_ conformer), together with its electron density surface, mapped as a function of the electrostatic potential. B) Optimized geometries of the ground state (S_0_) and the lowest singlet excited state (S_1_) in DMSO. All data are calculated at the PCM‐M06‐2X/def2‐TZVP level of theory. C) Jablonski diagram for the *C*_s_ DTTO conformer in DMSO, calculated at the STEOM‐DLPNO‐CCSD level, using the previously mentioned DFT optimized geometries; HOMO and LUMO are also reported since contributing more than 95% in the transitions between S_0_ and S_1_.

The structural and electronic properties of DTTO (**Figure** **2**A) were investigated in DMSO with the aid of DFT calculations at the M06‐2X/def2‐TZVP level of theory (see Experimental Section for further details). In its ground state (S_0_), the fully optimized DTTO molecule exhibits two virtually isoenergetic minima (belonging to the C_2_ and *C*_s_ point groups, ΔE ≈ 1 meV) which differ by the mutual orientation of the two peripheral phenyl moieties relative to the DTTO central core (Figure S3, Supporting Information). Both conformers display a completely planar core based on three fused thiophene rings, with the central one being oxidized to S,S‐dioxide, while the lateral phenyl rings are skewed by an angle of ≈41° (Figure 2B and S3, Supporting Information). Notably, regardless of the conformer, DTTO displays a very strong permanent electric dipole moment of 5.64 D, due to the presence of the strong electron‐accepting S,S‐dioxide group (Figure 2A), providing a significant driving force for aggregation.^[^ ^35^, ^36^ ^]^

Regarding the electronic properties of DTTO, both the highest occupied molecular orbital (HOMO) and lowest unoccupied molecular orbital (LUMO) are mostly centered on the fused thiophene core (Figure 2C), with some degree of delocalization onto the peripheral phenyl moieties observed in the upper‐ or lower‐lying frontier molecular orbitals (Figure S3, Supporting Information). Nevertheless, it should be emphasized that such orbitals are well separated from the HOMO and LUMO, so the photophysics of DTTO in the visible and near‐UV region is expected to be dominated by just the HOMO and LUMO.

As inferred from molecular‐orbital analysis, TD‐DFT calculations estimate an S_0_ → S_1_ transition, having a pure HOMO → LUMO character and a high oscillator strength (*i.e.*, *f* = 0.65), with well‐separated upper‐lying singlet excited states at more than 1.4 eV above S_1_ (Table S1 and S2, Supporting Information). Consequently, only S_1_ is expected to play a significant role in the photophysics of DTTO.

The energy of the vertical S_0_ → S_1_ transition is estimated at 3.31 eV (i.e., 374 nm) for both the conformers at the M06‐2X TD‐DFT level, which is slightly overestimated if compared to the experimental absorption maximum found at 3.04 eV (i.e., 408 nm). To improve the theoretical description, coupled‐cluster calculations at the STEOM‐DLPNO‐CCSD level were also performed, successfully locating the S_0_ → S_1_ excitation at 3.08 eV (i.e., 403 nm), in excellent agreement with the experiment (Table S3, Supporting Information). However, except for the mere energy value of the excitations, higher‐level post‐HF calculations confirm the scenario depicted by TD‐DFT (compare Table S2 and S3, Supporting Information). Indeed, the S_0_ → S_1_ transition dipole moment is always calculated to be oriented parallel to the phenyl–phenyl molecular axis (Figure 2B), with an intensity of 7.19 or 6.19 Debye, if calculated at the TD‐DFT or STEOM‐DLPNO‐CCSD level.

Upon relaxation of the lowest excited state, TD‐DFT calculations estimate that the dihedral angle between the planar DTTO core and the nearby phenyl rings passes from 41° in the ground state to just 25° in the fully optimized S_1_ state, with a shortening of the inter‐ring C—C bond, indicating a higher double‐bond nature in such excited state (Figure 2B). Assuming full relaxation and isotropic photon emission from randomly oriented molecules, the radiative rate constant for the S_1_ → S_0_ transition can be calculated according to the equation

(1) $$k_{r}=\frac{16\pi^{3}}{3\varepsilon_{0}h}{\tilde{\nu }}^{3}{\left|\vec{\mu }\right|}^{2}$$

where $\tilde{\nu }$ is the S_1_–S_0_ energy gap in wavenumber and *μ* is the dipole moment associated with the transition, resulting in a *k*_r_ = 1.84 · 10^8^ s^−1^ and a radiative lifetime of 5.4 ns. Such a theoretical estimate is in line with the experimentally determined radiative lifetime of (6.3 ± 0.3) ns, calculated for the DTTO single molecule in DMSO solution, based on the related PLQY and lifetime values (see previous section).

### 2.3 DFT Investigation on DTTO Dimers

To semiquantitatively explore the structural and electronic properties of DTTO in its possible aggregated forms, a simple dimeric model was considered. The main driving forces considered for aggregation include: 1) the strong permanent dipole–dipole interactions between DTTO molecules; 2) the *π*‐stacking of their aromatic units; and 3) the formation of other intermolecular interactions, like hydrogen bonds.

**Figure 3:**
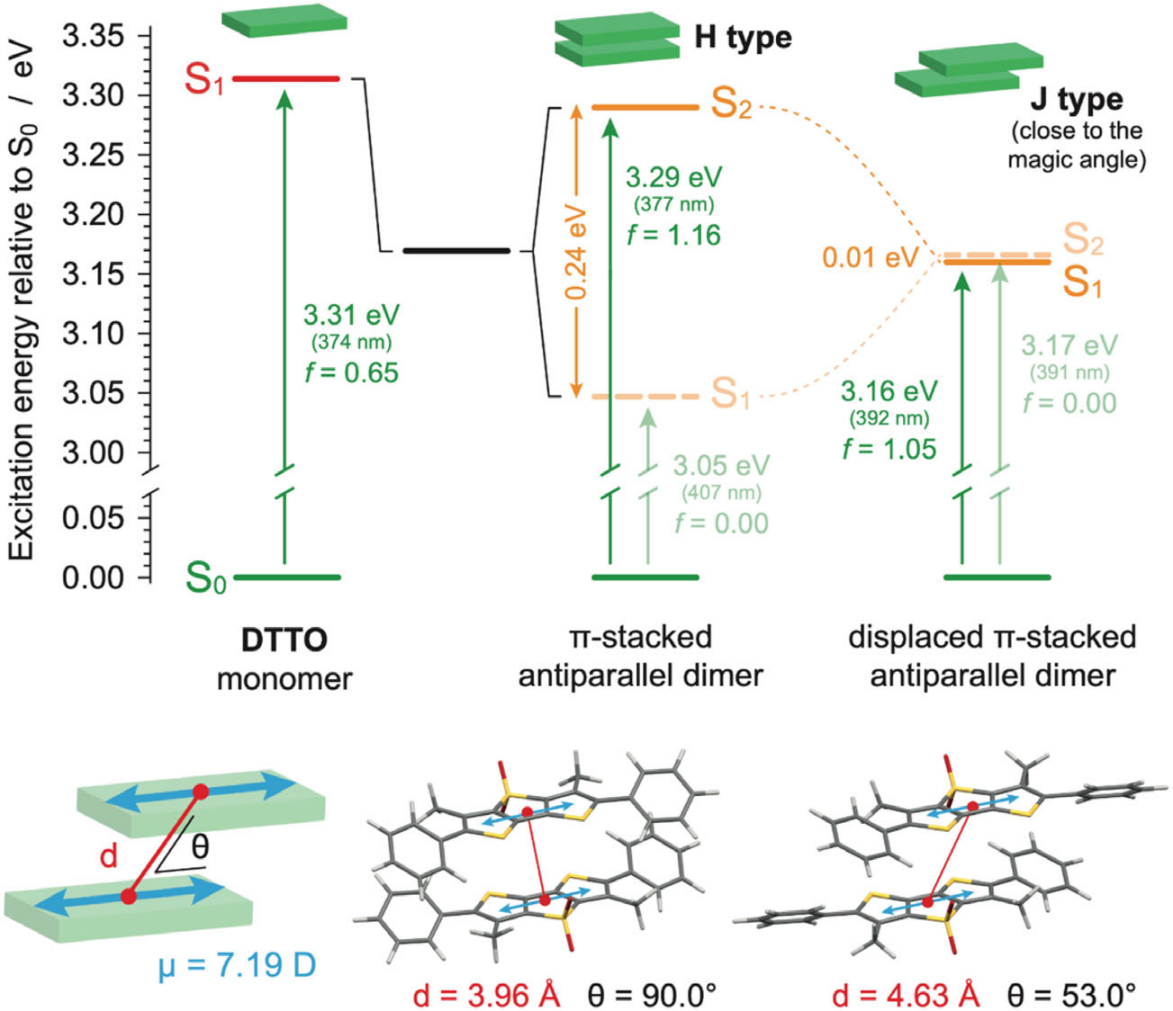
Excited‐state scenario of DTTO single molecule and two possible dimeric configurations, computed by TD‐DFT on fully optimized ground‐state structures. Dark states are marked with transparent dashed lines (top). Geometrical parameters used in Frenkel exciton theory of molecular H‐ and J‐aggregates are also reported (bottom).

The most stable dimeric geometry was found to adopt an H‐type antiparallel π–π stacked conformation, having a *C*_2h_ point‐group symmetry. This arrangement is primarily driven by the strong attraction between the large permanent dipole moments of the DTTO molecules, as well as favorable π–π interactions between the monomers (**Figure** **3**). This aggregate is predicted to be virtually nonemissive, having an energy splitting of 0.24 eV between the in‐phase and out‐of‐phase combinations of the transition dipole moments associated with the S_0_ → S_1_ transition of the DTTO single molecules (Table S5, Supporting Information).^[^ ^37^ ^]^ Notably, the bright excitation (i.e., S_0_ → S_2_ in this dimer) is calculated to be nearly isoenergetic with the S_0_ → S_1_ transition of the monomer, differing by only 0.02 eV (Figure 3).

The energy splitting resulting from TD‐DFT calculations is in line with that computed using Equation (2), considering the optimized geometry of the dimer (Figure 3), the transition dipole moment (*μ*) of the DTTO monomer, and the dielectric constant of a general polar aromatic environment (*ε*_r_ ≈ 4.5), as expected in a DTTO aggregate.

(2) $$J_{C}=\frac{\mu^{2}\left(1-3cos^{2}\theta \right)}{4\pi \varepsilon_{0}\varepsilon_{r}R^{3}}$$

Dimer screening also revealed a second pair of virtually isoenergetic structures, positioned just 0.10 eV above the H‐type dimer (Figure S4A, Supporting Information). In both conformations, the two DTTO units are slipped to a different extent along the virtual phenyl–phenyl axis, maximizing a double C—H···O=S intermolecular interaction between the peripheral phenyl groups and the central SO_2_ moiety (Figure S4B‐C, Supporting Information). Such deformation lowers the *C*_2h_ point‐group symmetry of the H‐type dimer to *C*_i_ when maintaining the antiparallel π‐stacked conformation of the DTTO units (M‐type), or to *C*_2_ when aligning their permanent dipole moments (J‐type) (see Figure S4, Supporting Information, for details). In a bulk aggregate, the latter arrangement would lead to an unfeasible build‐up in charge distribution, unless an identical parallel‐displaced stack is paired with its dipole moment oriented in the opposite direction. Consequently, for simplicity, only the displaced π‐stacked antiparallel dimer is shown in Figure 3. In this dimer, the *θ* angle—defined by one of the two parallel S_0_ → S_1_ transition dipole moments and the displacement vector *d* between the two DTTO units—is 53.0°, just 1.7° lower than the magic angle.^[^ ^38^ ^]^ As a result, a very low exciton coupling can be expected (*J*_C_ ≈ −0.006 eV, Equation (2)), in agreement with TD‐DFT, results which show nearly degenerate S_1_ and S_2_ states in the Franck–Condon region, with the bright state slightly lower in energy than the dark one (Table S6, Supporting Information). It should be stressed that, despite the negligible coupling, a 0.15 eV redshift for the S_0_ → S_1_ transition is estimated for this dimer compared to the monomeric DTTO molecule (Figure 2, Table S2 and S6, Supporting Information).

Finally, a T‐type DTTO dimer was also examined (Figure S4D, Supporting Information). In this configuration, the monomeric units interact weakly through peripheral phenyl groups and methyl substituents on the DTTO core, resulting in a negligible electronic coupling between the monomers. Thus, this dimer is only 0.24 eV more stable than two noninteracting monomers and remains 0.48 eV higher in energy than the *C*_2h_ H‐type dimer.

### 2.4 PL Interpretation

According to the experimental results, the radiative lifetime of DTTO in DMSO solution is $\tau_{\text{RAD}}=\frac{\tau }{\mathrm{PLQY}}$ = (6.3 ± 0.3) ns, considering the spread in PLQY values. Experimental $\tau_{\text{RAD}}$ is thus consistent with the theoretically computed *τ*_RAD_ = 5.4 ns. Quick precipitation in water forms H‐type aggregates whose PL decay kinetics show two well‐separated time scales, namely *τ*_S_ = 0.4 ns [*A*_S_ = 0.52] and *τ*_L_ = 5.7 ns [*A*_L_ = 0.48]. The emission is dominated by the long‐lived component since the time‐integrated contribution of the short‐lived component is negligibly small (10^−4^). We conjecture that the emitting species are DTTO molecules dissolved in a DMSO wetting layer coating the crystal structure. This rationalizes the spectral shape and lifetime that coincide with that of the DTTO monomer. Specifically, observing the PL emissions at longer delays, the tail of the dynamics of DTTO in DMSO and water perfectly overlaps (Figure S5, Supporting Information). The estimated total PLQY from the sample in water is 1%–2%. Considering that the PLQY of DTTO molecules in DMSO is about 0.9, we conclude that about 2% of the DTTO molecules are dispersed in the adsorbed layer at the crystal surface, while the crystals themselves are virtually nonemitting, according to our theory.

The PL spectrum of the DTTO fibers has the same line shape as DTTO in DMSO, albeit slightly redshifted, and almost overlaps with that of aggregates in water. PL lifetime is, however, strongly reduced, exhibiting a predominant fast exponential decay *τ*_1_ = 350 ps (A_1_ = 0.9), followed by a small tail with time constant *τ*_2_ = 5.7 ns (A_2_ = 0.1). The PLQY is 0.3, and the corresponding *τ*_RAD_ is (2.7 ± 0.7) ns. Based on these numbers, radiationless deactivation alone cannot explain the shorter photoluminescence (PL) lifetime. This suggests that the excited state in the fiber is an efficient emitter, consistent with the J‐like coupling found by theoretical studies. The dipole moment of the optically allowed J‐aggregate is described by Equation S2, Supporting Information. Considering Equation S2, Supporting Information (describing the magnitude of the transition dipole moment of an aggregate of N monomers, see Supporting Information for further details) and plugging in the S_0_ → S_1_ transition dipole moment of the DTTO single molecule computed at the STEOM‐DLPNO‐CCSD level (*μ* = 6.19 D, see previous section) and *n* = 2 (i.e., two monomeric units, as for the model J‐dimer of Figure 3), the resulting radiative lifetime (*τ*_RAD_ = 1/*k*_r_, calculated by Equation (1)) shortens to 2.5 ns, which is in excellent agreement with the value estimated experimentally (*i.e.*, *τ*_RAD_ = *τ*/PLQY = (2.7 ± 0.7) ns, see above).

PL polarization anisotropy decay, shown in Figure S6, Supporting Information, indicates that DTTO monomers and small aggregates exhibit very similar behavior, characterized by a single‐exponential decay with a time constant *τ* = 240 ps, attributed to molecular rotation. In water samples, this decay is associated with the fraction of isolated monomers. Notably, in aggregates, the initial anisotropy value is lower, suggesting rapid intraaggregate relaxation. In contrast, fibers behave differently: their anisotropy signal persists for several hundred picoseconds, with the initial anisotropy exceeding 0.4, suggesting crystalline order.

The complex nature of the emission from aggregates in water also appears from the spectral evolution of the PL at different time delays. As depicted in Figure S7, Supporting Information, the spectra of DTTO in DMSO and fibers remain unchanged over time, while that of DTTO in water shifts toward lower energies. This points to the presence of different emitting species, with the monomer dominating at longer delays. The redshift observed in water with respect to the monomer in DMSO can be rationalized by the different solvent polarity (see Supplementary Information for details: Figure S8 and Equation S3, Supporting Information).

### 2.5 Transient Transmission

**Figure 4:**
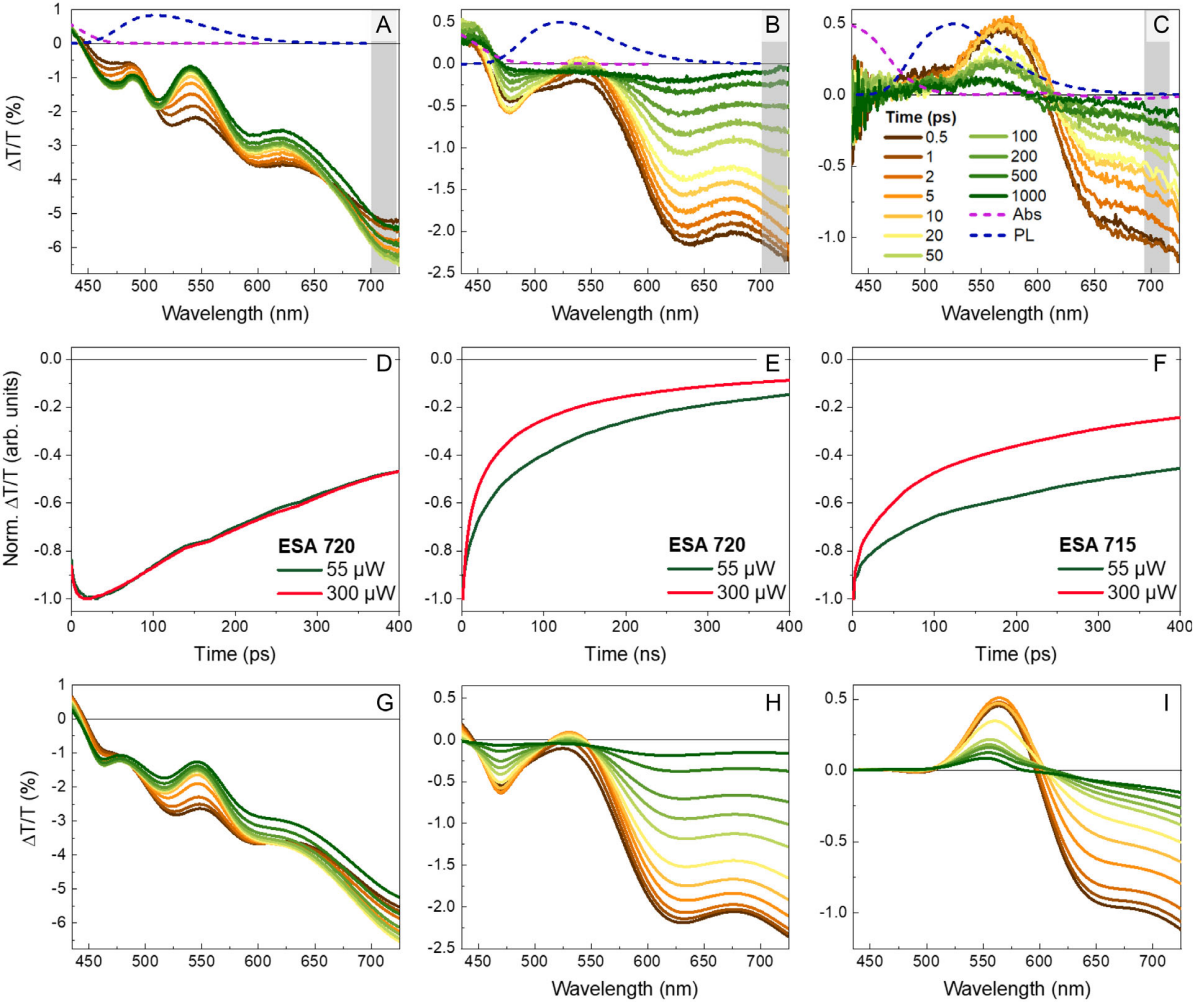
Transient transmission spectra over time of DTTO in A) DMSO, B) water, and C) fibers. The time evolution is indicated by the color gradient of the line (from brown = 0.5 ps to green = 1000 ps). Dashed lines represent absorption and PL spectra measured in continous wave. Normalized TA signal decay, measured at 780 nm for DTTO in D) DMSO, E) water, and F) DTTO fibers, respectively, measured at different power densities. Predicted pump‐probe spectra of DTTO in G) DMSO, H) water, and I) fibers, respectively, measured at different time delays.

**Figure** **4** reports the transient transmission difference (ΔT/T) spectra of DTTO monomer in DMSO, DTTO aggregates in water, and DTTO cell‐made fibers in water aqueous suspension after pumping the sample with a 60 μW laser pulse, 100 fs time duration, at 400 nm. The ΔT/T spectra of DTTO monomers in DMSO exhibit an isosbestic point at about 450 nm (Figure 4A). The positive ΔT/T signal observed at wavelengths below this point is assigned to ground‐state bleaching (GSB), while the negative ΔT/T signal at wavelengths >450 nm is assigned to excited state absorption (ESA). The broad ESA band features distinct peaks at 475, 520, 590, and 720 nm, which evolve over time. During the first 10 ps, a faster decay is observed between 500 and 600 nm, along with a concurrent build‐up at 475 nm and 720 nm (Figure S9, Supporting Information). The faster decay develops a sort of dip in the spectrum, which we assigned to the growth of stimulated emission (SE, ΔT/T > 0), which, however, never overtakes ESA (ΔT/T < 0). In contrast, the build‐up dynamics are associated with a redshift of the involved ESA peaks.

Once relaxation is completed, the ΔT/T spectrum decays without changing shape on the nanosecond timescale, as reported in Figure S9, Supporting Information. Numerical fitting of the kinetics yields again a quasi‐single‐exponential decay with a time constant *τ*_2_ = 5.7 ns and a small faster decay component *τ*_1_ = 150 ps (Table S7, Figure S9B, Supporting Information). These values closely match the PL lifetime previously reported, further supporting the assignment of the observed dynamics to the lowest singlet excited state of DTTO.

Transient data provide additional information on the excited‐state dynamics that fit well with the picture so far reported. The experimental ESA features correlate well with the energy differences between S_1_ and upper‐lying singlet excited states calculated by STEOM‐DLPNO‐CCSD for DTTO in its fully relaxed S_1_ minimum‐energy geometry (Figure 2C). Three excitations from S1 dominate the spectrum: i) the intense S1 → S3 transition at 1.66 eV (*i.e.*, 746 nm); ii) two nearby excitations to S5 and S6 at 2.00 and 2.11 eV (i.e., 622 and 587 nm), respectively (Figure 2C and Table S4, Supporting Information).

DTTO aggregates in water show very different ΔT/T spectra with respect to the monomer (Figure 4B). The isosbestic point is shifted to a longer wavelength, about 475 nm, consistent with the broadening of the absorption spectrum, while SE is not evident since it appears to be overwhelmed by ESA. There are two ESA peaks, at 630 nm and 725 nm, exhibiting different lifetimes and thus most probably related to two different excited states. We note that the initial dynamics seen in the monomer and assigned to planarization are not observed, consistently with the expected steric hindrance in aggregates (Figure S9E, Supporting Information). Furthermore, the excited‐state deactivation is much faster in aggregates, likely due to additional relaxation pathways created by intermolecular interactions, consistent with time‐resolved PL measurements (Figure S9H, Supporting Information).

Turning to ΔT/T spectra of fibers (Figure 4C), we observe a clear SE signal around 550 nm, while ESA appears at wavelengths longer than 620 nm. Notably, two ESA peaks are observed at 640 nm and 725 nm, like aggregates in water, again characterized by different lifetimes that suggest two distinct excited states. The excited‐state deactivation in fiber is much faster than that in monomer and similar to that observed in aggregates in water. Apparently, in spite of different molecular packing, there are similar nonradiative adiabatic transitions.

The pump power dependance of the ESA signal further highlights solid‐state effects in aggregates (Figure 4D–F). While the monomer does not show any change in kinetics upon sixfold increasing pump intensity, ESA decay in water and fibers gets markedly faster at higher pump intensity. We assign this to a bimolecular deactivation process that implies energy migration and intermolecular interactions. The bimolecular decay rate, however, differs in the two samples, as it is affected by the migration process occurring in different topologies.

Interestingly, ΔT/T spectra of fibers do not show any spectral signature of GSB, expected at wavelengths shorter than 500 nm (see Figure 1 for the absorption edge). Obviously, processes such as SE or ESA stem from excited‐state population and consequently depletion of the ground state. Therefore, it is not possible to have an SE or ESA signal without the appearance of GSB. To account for this discrepancy, we conjecture that scattering plays a crucial role in transient spectra.

To support this hypothesis, we developed a simplified model of the complex dielectric function that allows a unified description of the different data, using a set of classical oscillators with pump‐dependent oscillator strength (see Supplementary Information for details). Using the imaginary part of the dielectric function, we could successfully reproduce the measured ΔT/T spectra and kinetics of monomers in solution and water (Figure S10, Supporting Information) according to the interpretation discussed above. To reproduce spectra from the fibers, we assume that the ΔT/T signal includes the pump‐induced modulation of scattering. The scattering cross section (Equation (3)) in the ground (*G*) and excited (X) state depends on the complex dielectric function^[^ ^39^ ^]^

(3) $$scatt_{G,X}=\omega^{3}{\left|\varepsilon_{G,X}-1\right|}^{2}$$

where $\varepsilon_{G,X}$ are built following the same approach used for DTTO monomers.

Figure 4I shows simulations of the time‐dependent spectra obtained by accounting for both absorption and scattering contributions (Equation S6, Supporting Information). From the simulations, we understand that aggregates and fibers have similar electronic structures and that they have an additional state compared to the monomer.

**Figure 5:**
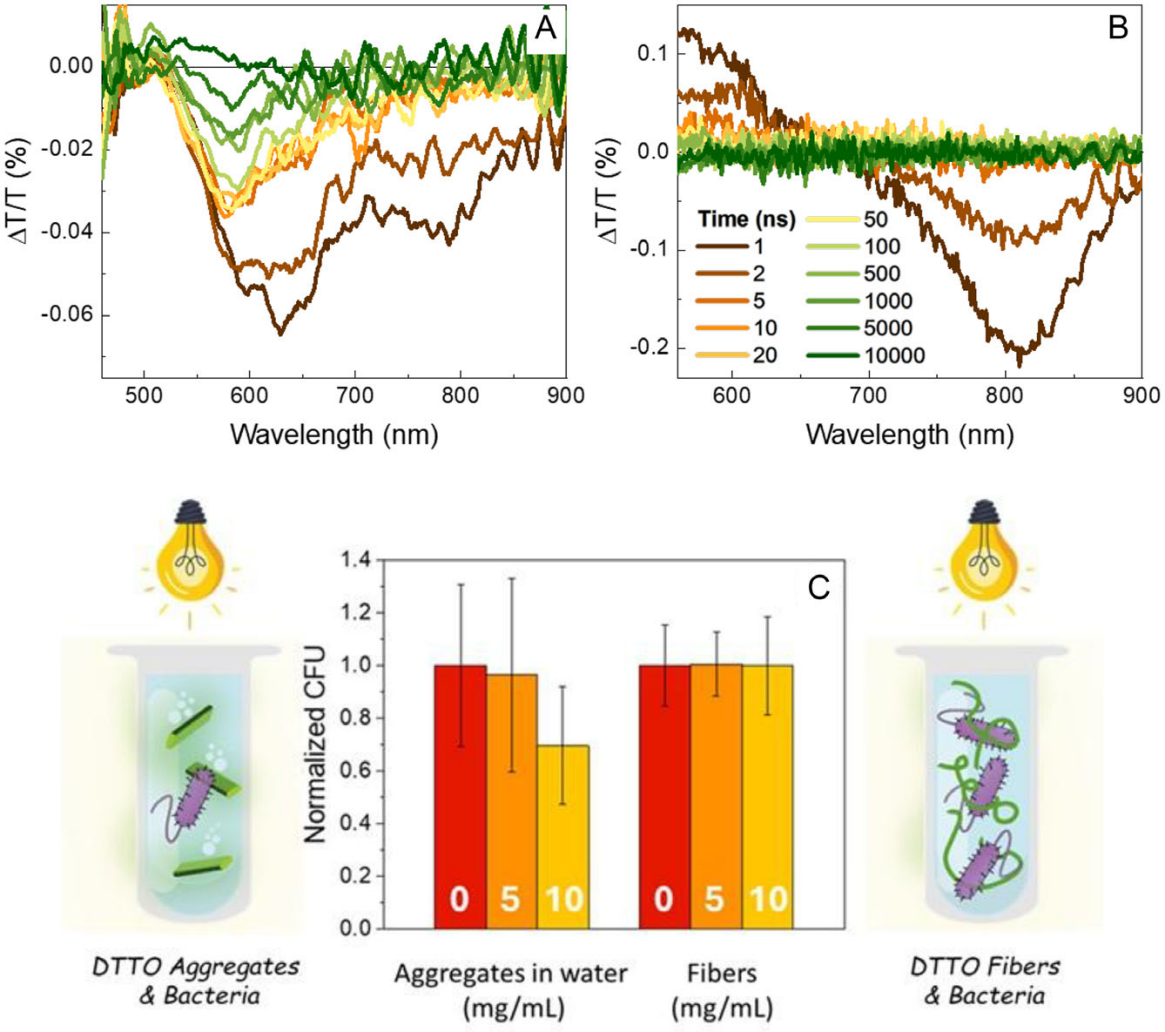
Transient transmission spectra over time of A) DTTO in water and B) DTTO fibers at long delays. The time evolution is indicated by the color gradient of the line (from brown = 1 ps to green = 10). C) Histogram representing the biocidal activity of DTTO aggregates formed in water (left) and DTTO fibers (right) at different concentrations (0–10 μg mL^−1^) against *E. coli* under light conditions. Data are expressed as mean ± SD. H_2_O; 0 μg mL^−1^: 1 ± 0.3062 (*N* = 8), 5 μg mL^−1^: 0.9636 ± 0.3675 (*N* = 8), 10 μg mL^−1^: 0.6950 ± 0.2239 (*N* = 8). Fibers; 0 μg mL^−1^: 1 ± 0.1524 (*N* = 10), 5 μg mL^−1^: 1.004 ± 0.1206 (*N* = 8), 10 μg mL^−1^: 0.9982 ± 0.1859 (*N* = 10). Illumination cycles were conducted by exposing the colonies to 470 nm light for 10 min, followed by 10 min under dark conditions (3 times).

In the very long time scale, water aggregates’ spectra show a further band around 580 nm, which emerges at 5 ns pump‐probe delay and persists up to the microsecond range, as shown in **Figure** **5**A. This band may arise either from the relaxation of the excited state associated with the ESA band initially observed at 620 nm or from the formation of a new electronic state. Interestingly, in the spectra of fibers—which display a similar spectral signature in the subnanosecond regime—no evidence of such a long‐lived state is observed (Figure 5B and S11, Supporting Information). Given that this long‐lived feature is also absent in monomer spectra, we exclude triplet‐triplet transitions and instead speculate that it arises from a charge‐transfer state enabled by specific molecular packing (Figure S12, Supporting Information).

Due to the long lifetime, such a state could interact with the environment, leading to the formation of radicals or other reactive species that may compromise photostability and induce phototoxicity in living systems. Accordingly, the absence of a long‐lived state in DTTO fibers may offer an advantage over aggregates for bioapplications. To investigate this effect, *E. coli* in suspension were exposed to either DTTO fibers or aggregates at concentrations of 5 and 10 μg mL^−1^ (Figure 5C and S13). The samples were then subjected to continuous illumination with 488 nm light for 30 min. Potential phototoxic effects of DTTO in its aggregated forms were assessed using minimum inhibitory concentration (MIC) experiments. In these experiments, the reduction in colony‐forming unit (CFU) count compared to control conditions would indicate an antimicrobial or cytotoxic effect, likely due to reactive species generated upon illumination. The results are shown in Figure 5C, where CFU counts are normalized to the control samples, which consisted of bacteria exposed to either DMSO or 1% Triton, the media used to disperse DTTO and its fibers. Interestingly, while DTTO aggregates formed in water exhibit significant phototoxicity, the fibers do not show any measurable reduction in CFU count under the same conditions. This finding nicely supports our hypothesis that the absence of long‐lived excited states in the fibers, arising from their distinct molecular packing and morphology, effectively prevents the generation of harmful reactive species upon illumination, making them suitable photostable biomaterials for bioapplications.

## 3 Conclusions

In this study, we employed time‐resolved spectroscopy in combination with quantum‐chemical modeling to study the excited‐state dynamics of DTTO aggregates, with a particular focus on the unique properties of bioproduced fibers assembled within living cells.^[^ ^22^, ^23^, ^24^ ^]^

The results presented here indicate that DTTO self‐assembled fibers are stabilized by noncovalent interactions, which, although insufficient to promote extended electronic delocalization, still support energy transport through weak intermolecular interactions, as evidenced by second‐order decay kinetics. This behavior suggests that charge transport along the fiber axis may also be possible.^[^ ^24^ ^]^

To account for the observed fibers’ emission yield, we propose that weak coherent coupling gives rise to emissive J‐type dimers, likely oriented along the fiber axis, as supported by DFT calculations. Owing to the relatively weak coupling strength, much smaller than the disorder‐induced spectral broadening, steady‐state spectral line shapes do not exhibit distinct excitonic features. However, we propose that exciton dimers renormalize the radiative decay rate, thereby explaining the higher PLQYobserved in fibers compared to the DTTO aggregates in solution.

Additionally, transient spectroscopy measurements on the microsecond timescale reveal that the fibers do not support long‐lived excited states, in contrast to DTTO aggregates produced in an aqueous environment. Given that such states are often associated with high chemical reactivity, the phototoxicity of DTTO in its aggregated forms was evaluated. Aggregates formed in water exhibited light‐induced phototoxicity in bacterial systems, likely due to the generation of reactive oxygen species, whereas cell‐made fibers did not display any measurable toxicity under comparable conditions. These findings suggest that intracellular self‐assembly imposes a peculiar molecular packing and morphology, which not only modulates the optical properties of DTTO but also significantly alters its interactions with biological systems, making the fibers a safer and more suitable platform for applications in bioelectronics or intracellular delivery. Interestingly, the excited‐state transmission spectra of the fibers display a clear SE peak, suggesting the possibility of optical gain, assuming that some degree of optical guiding is present. These properties could be particularly relevant for labeling applications in cells.^[^ ^40^ ^]^

In conclusion, DTTO fibers represent a novel class of cell‐made biomaterials, characterized by unique structural and functional properties that can be directly harnessed in biological contexts. Their spontaneous formation in living cells, combined with favorable electro‐optical and biocompatibility characteristics, makes them strong candidates for signal transduction applications aimed at monitoring cellular processes under diverse physiological conditions. Our findings align with recent trends in organic bioelectronics, which emphasize the importance of soft, functional materials for efficient coupling with biological environments.^[^ ^20^, ^41^ ^]^

## 4 Experimental Section

4.1

4.1.1

##### Cell Culture Maintenance

In vitro experiments were conducted using C2C12 (mouse myoblasts) cells, both obtained from ATCC. The cells were maintained in T‐25 culture flasks with Dulbecco's modified Eagle medium high glucose (DMEM‐HG), supplemented with 10% heat‐inactivated fetal bovine serum (FBS) and 1% GlutaMAX (0.5 mM, Invitrogen). Cultures were kept in a humidified incubator at 37 °C with 5% CO_2_. Upon reaching confluence, cells were detached using a 2 × trypsin‐EDTA solution, seeded onto sterilized substrates, and allowed to grow for at least 24 h before experiments were performed. To enhance cellular adhesion, the substrates were precoated with a fibronectin layer (2 μg mL^−1^ in phosphate‐buffered saline (PBS)) and incubated at 37 °C for a minimum of 1 h. For aligned cell seeding, a microcontact printing technique was employed, as detailed in the following section.

##### Cell Seeding and DTTO Fiber Formation

C2C12 cells were seeded at a density of ≈20 000 cells cm^−2^ in a 12‐well tissue culture plate, with 1 mL of complete culture medium per well. DTTO–synthesized according to reference^[^ ^22^ ^]^ – was initially dissolved in DMSO at a concentration of 2.5 mg mL^−1^ to create a stock solution, which was then diluted in DMEM to a final concentration of 25 mg mL^−1^ before being added to the cells. The cells were incubated at 37 °C in a 5% CO_2_ atmosphere with 95% relative humidity for 1 h. Following the incubation, the remaining dye in the solution and any DTTO aggregates that failed to penetrate the cell membrane were removed by washing with PBS before proceeding with each experiment.

##### Confocal Imaging

Confocal imaging was performed using an inverted confocal laser microscope, Nikon Eclipse Ti2, with image acquisition managed by NIS‐Element Nikon Imaging Software. For localization experiments, cells were plated at a density of 20 000 cells cm^−2^ 24 h before the experiment. On the day of the experiment, the cells were incubated with DTTOs for 1 h. Following incubation, samples were washed with PBS to remove unbound molecules. Myotubes were differentiated and treated with DTTO as described in the previous sections. Subsequently, samples were incubated for 5 min with CellMask Deep Red (Thermo Fisher) to stain the plasma membrane. After washing with PBS, imaging was performed using a 60× objective to acquire Z‐stacks of consecutive confocal sections. DTTO and CellMask were excited using 403.3 nm (detection channel 500–550 nm) and 640 nm (collection channel 663–743 nm) lasers, respectively. The acquired images were analyzed using Fiji (ImageJ).

##### DTTO Fiber Isolation

Fibers were isolated from cells using a method similar to that described by Palamà et al.^[^ ^22^ ^]^ DTTO fibers were purified from whole cell lysates prepared in 50 mM Tris HCl (pH 7.4), 1% Triton X‐100, 5 mM EDTA, 150 mM NaCl, 1 mM Na_3_VO_4_, 1 mM NaF, 1 mM PMSF, 10 μM benzamidine‐HCl, 10 μg mL^−1^ aprotinin, 10 μg mL^−1^ leupeptin, and 10 mg mL^−1^ pepstatin A. In particular, the cells were detached from the substrate, suspended in lysis buffer, and washed several times by centrifugation (1550 rpm, 20 min, 4 °C) by removing the supernatant and resuspending in fresh lysis buffer after each centrifugation. The resulting solution was then stored at −80 °C for future use. As previously reported, this isolation process does induce changes in the fibers’ properties.^[^ ^24^ ^]^

##### DTTO Aggregates Formation in H_*2*_ *O*

DTTO aggregates in water were prepared by diluting a DMSO stock solution (2.5 mg mL^−1^) into Milli‐Q water to a final concentration of 25 mg mL^−1^, with a final DMSO content of 1% v/v. Samples were measured as soon as they were prepared to keep the aggregation extent and were freshly prepared for each experiment to maintain the extent of aggregation constant throughout the different experiments.

##### Steady‐State Absorbance Measurements

UV–Vis absorption measurements were conducted using two advanced spectrophotometers: a Varian Cary 5000 and a Perkin Elmer Lambda 1050, both equipped with deuterium lamps (covering the 180–320 nm range) and tungsten lamps (320–3300 nm). These instruments featured a monochromator and three detectors for enhanced sensitivity: a photomultiplier tube (180–860 nm), an InGaAs detector (860–1300 nm), and a PbS detector (1300–3300 nm). The absorption spectra were normalized by taking reference spectra under three conditions: 100% transmission (without the sample), 0% transmission (with an internal shutter to block light), and using the reference solvent in which the sample was dissolved.

##### Time Resolved Photoluminescence (TRPL)

TRPL measurements were performed using a Ti: Sapphire laser source (Chameleon Ultra II, Coherent), which produced pulses of 140 fs with a repetition rate of 80 MHz. Second harmonic generation was achieved using a barium borate crystal, and residuals of the fundamental were removed with two BG40 filters. The excitation beam (405 nm) was reflected by a dichroic mirror (LP425) before striking the sample. Emission was collected using an Acton SP2300i Princeton Instrument spectrograph coupled with a Hamamatsu C5680 streak camera and the Synchroscan sweep module. A Hamamatsu ORCA‐R2 C10600 CCD was used to record the streak image. Measurements captured the first 2 ns of decay with a temporal resolution of ≈20 ps.

For measurements on living cells and isolated fibers (TRPL microscopy), the excitation beam was collected with a 63X water immersion objective (for living cells) or a 40X objective (for other measurements) and focused onto the sample to ensure spatial resolution, achieving an excitation spot diameter of 1 μm and a power of 2 μW. The microscope field was observed via a flip mirror and CMOS camera (ORCA‐Flash 2.8, Hamamatsu), enabling precise focusing of the excitation beam using a sample XYZ differential micrometer translation stage. The emission signal was directed to the entrance slit of the spectrograph, as described earlier. For PL anisotropy measurements, a linear polarizer was placed between the sample and detector, enabling the collection of only the component of emitted light with polarization parallel/orthogonal (depending on the polarizer orientation) to the excitation laser polarization.

##### Transient Absorption (TA) Measurements

TA experiments were performed using a regeneratively amplified Ti:sapphire laser system (Coherent Libra II) as the light source, emitting 100 fs pulses centered at 800 nm with a 1 kHz repetition rate and an average power of 4 W. The pump pulses were generated using a frequency‐tunable noncollinear optical parametric amplifier (NOPA), capable of producing 100 fs narrowband pulses across a range of wavelengths from 480 nm to 1.6 μm. For the experiments in this study, the NOPA was pumped at 400 nm by the second harmonic of the Ti:sapphire laser, which was generated in a 2‐mm‐thick beta‐barium borate (BBO) crystal and seeded by a white‐light continuum (WLC), generated in a 1‐mm‐thick sapphire plate. This WLC was then spectrally filtered with an interference filter centered at 500 nm with a 10 nm bandwidth. The average pump power for the experiments was set to 50 μW. The seed pulse from the WLC was amplified in a 2‐mm type‐1 BBO crystal. To generate the broadband probe pulse, a portion of the fundamental beam was focused into a 2‐mm‐thick sapphire crystal, producing a WLC. The fundamental was then filtered out using BG39 filters, yielding probe pulses in the visible range (420–730 nm). The pump‐probe delay was precisely controlled using a motorized delay stage, and the transmitted probe signal was collected using an optical multichannel amplifier. This setup enabled the detailed investigation of transient absorption events in the samples.^[^ ^42^ ^]^

For the microsecond delay transient absorption measurements, the probe pulse was sourced from an amplified femtosecond laser (Light Conversion Pharos), which generates 280 fs pulses centered at 1030 nm. A broadband white‐light probe was produced by focusing the light onto a 1 mm sapphire plate. The pump pulses, with a duration of 1 ns and centered at 532 nm, were provided by the second harmonic of a Q‐switched Nd:YVO4 laser (Innolas Picolo), which was electronically triggered and synchronized with the femtosecond laser via an electronic delay generator. The experiments were conducted with a repetition rate of 2 Hz and an average pump power of 3.5 mW. The detection system used was similar to the one described earlier.

##### MIC Assay

MIC experiments were performed to evaluate the antimicrobial activity of DTTO in different conditions. *E. coli* cultures were grown overnight in LB medium at 37 °C with shaking (200 rpm) until reaching the exponential phase. The bacterial suspension was then diluted to an optical density at 600 nm (OD600) of ≈0.01 in fresh LB medium. Bacteria were exposed to either DTTO fibers (previously isolated from C2C12 cells) or DTTO molecular aggregates, which were obtained by diluting DTTO from DMSO into water, leading to immediate aggregation. Control samples were prepared by exposing bacteria to equivalent volumes of DMSO or Triton X‐100, the dispersing media for DTTO and DTTO fibers. The samples were incubated at different concentrations of DTTO under standard growth conditions. To assess phototoxicity, a subset of samples was illuminated with 488 nm light for 30 min (10 min illumination, 10 min in dark, repeated 3 times), while nonilluminated controls were kept in the dark. After incubation, serial dilutions were plated onto LB agar and incubated overnight at 37 °C. CFU were counted the next day and normalized to the control samples to determine the antimicrobial effects.

##### Computational Details

DFT calculations were carried out using the B.01 revision of the Gaussian16 program package,^[^ ^43^ ^]^ in combination with the M06‐2X hybrid meta exchange‐correlation functional,^[^ ^44^, ^45^ ^]^ which has been specifically designed to work well with charge‐transfer excitations having intermediate spatial overlap.^[^ ^46^ ^]^ The def2‐TZVP basis set was adopted for all atoms,^[^ ^47^ ^]^ and the polarizable continuum model (PCM) was employed to consider dimethyl sulfoxide (DMSO) solvation effects.^[^ ^48^, ^49^ ^]^ All calculations on the DTTO single molecule were performed within both the C_2_ and C_s_ point‐group symmetry, resulting in virtually isoenergetic minima (ΔE ≈ 1 meV, in the ground state). TD‐DFT calculations, at the same level of theory used for ground‐state optimizations, were used to compute Franck‐Condon excitations (within the PCM linear‐response formalism) and to fully optimize the lowest‐energy excited state (S_1_) of the DTTO single molecule in solution (using a PCM state‐specific approach).^[^ ^50^ ^]^ Analytical frequency calculations at both DFT and TD‐DFT levels were always carried out to confirm the nature of the stationary points on the potential energy surfaces.

To further explore the excited‐state properties of single‐molecule DTTO at a higher level of theory, STEOM‐DLPNO‐CCSD single‐point calculations on the S_0_ and S_1_ minimum‐energy geometries previously obtained by DFT (see above) were performed as implemented and recommended in ORCA 6.^[^ ^51^ ^]^ The STEOM‐DLPNO‐CCSD approach exploits the full potential of the DLPNO (domain‐based local pair natural orbital) approximation to reduce the computational scaling of a standard STEOM‐CCSD method, while keeping the same accuracy of such coupled‐cluster method.^[^ ^52^, ^53^ ^]^ In brief, such approach employs a DLPNO approximation to perform ground‐state CCSD efficiently, followed by a similarity‐transformed equation‐of‐motion CCSD (STEOM‐CCSD) step that compresses the excitation manifold into a compact CIS‐like subspace, thereby delivering near‐canonical accuracy in excited‐state energies at substantially reduced cost. Such calculations were performed using the def2‐TZVP(‐f) basis set,^[^ ^47^ ^]^ in combination with the def2‐TZVP/C and def2/J auxiliary basis sets, to take advantage of the resolution of identity approximation (i.e., density fitting).^[^ ^54^, ^55^ ^]^ The conductor‐like PCM (CPCM) was also considered to take into account DMSO solvation.^[^ ^56^ ^]^ The single‐point calculations on the S_1_ minimum‐energy geometries were also used to simulate the transient absorption spectrum of DTTO, considering S_1_ as the targeted excited state.

To shed light on the structural and electronic properties of the DTTO aggregates or fibers, possible conformations of DTTO dimers were fully optimized, starting from educated guesses, at the same level of theory used for single‐molecule DFT calculations (i.e., M06‐2X/def2‐TZVP in DMSO, using PCM as implemented in Gaussian 16, see above). Whenever possible, the use of symmetry was exploited to speed up the geometry optimizations and related frequency calculations. TD‐DFT calculations, at the same level of theory, have also been carried out to model the excited‐state scenario within the dimers. Both DMSO and water solvation environments were tested in TD‐DFT calculations (using linear‐response PCM), leading to comparable results; accordingly, only data in DMSO were reported for a better comparison with the DTTO single molecule in solution.

All the pictures of molecular orbitals and density surfaces were created in GaussView 6,^[^ ^57^ ^]^ molecular geometries and related overlaps and comparisons were accomplished using Mercury 2024.3.1.^[^ ^58^ ^]^ Natural transition orbital analysis was also used to obtain a compact representation of the vertical singlet excitations computed by both TD‐DFT and STEOM‐DLPNO‐CCSD methods.^[^ ^59^ ^]^

##### Statistical Analysis

Data were all expressed as mean ± SD. Normal distribution was assessed using the Shapiro Wilk normality test. All samples were compared with the control condition using the unpaired Student t‐test. The significance level was preset to p < 0.05 for all the tests. Statistical analysis was carried out using GraphPad Prism 9 software.

## Conflict of Interest

The authors declare no conflict of interest.

## Supporting information

Supplementary Material

## Data Availability

The data that support the findings of this study are available from the corresponding author upon reasonable request.

## References

[1] J. M. Lehn , PNAS 2002, 99, 4763. 11929970 10.1073/pnas.072065599 PMC122664

[2] P. J. Cragg , Supramolecular Chemistry: From Biological Inspiration to Biomedical Applications, Springer Science+Business Media B.V., Berlin and Heidelberg 2010.

[3] S. Chagri , D. Y. W. Ng , T. Weil , Nat. Rev. Chem. 2022, 6, 320. 37117928 10.1038/s41570-022-00373-x PMC8972907

[4] M. Dergham , S. Lin , J. Geng , Angew. Chem. Int. Ed. 2022, 61, e202114267. 10.1002/anie.202114267 35037350

[5] C. Liu , H. Ma , S. Yuan , Y. Jin , W. Tian , ACS Nano 2025, 19, 2047. 39779487 10.1021/acsnano.4c16669

[6] X. Zhang , J. Wang , Y. Zhang , Z. Yang , J. Gao , Z. Gu , Chem. Soc. Rev. 2023, 52, 8126. 37921625 10.1039/d2cs00999d

[7] N. Song , F. Tian , Y. Zou , Z. Yu , Interfaces 2024, 16, 45821. 10.1021/acsami.4c10653 39177358

[8] S. Kim , G. Park , D. Kim , M. S. Hasan , C. Lim , M. S. Seu , J. H. Ryu , Adv. NanoBiomed Res. 2024, 4, 2300137.

[9] M. Pieszka , S. Han , C. Volkmann , R. Graf , I. Lieberwirth , K. Landfester , D. Y. W. Ng , T. Weil , J. Am. Chem. Soc. 2020, 142, 15780. 32812422 10.1021/jacs.0c05261 PMC7499420

[10] Y. Chen , M. Zuo , Y. Chen , P. Yu , X. Chen , X. Zhang , W. Yuan , Y. Wu , W. Zhu , Y. Zhao , Nat. Commun. 2023, 14, 5229. 37634028 10.1038/s41467-023-40935-1 PMC10460442

[11] E. V. Amadi , A. Venkataraman , C. Papadopoulos , Nanotechnol. 2022, 33, 132001. 10.1088/1361-6528/ac3f54 34874297

[12] J. D. Hartgerink , E. Beniash , S. I. Stupp , PNAS 2002, 99, 5133. 11929981 10.1073/pnas.072699999 PMC122734

[13] G. Albino de Souza , F. de Castro Bezerra , T. D. Martins , ACS Omega 2020, 5, 8804. 32337442 10.1021/acsomega.0c00381 PMC7178805

[14] D. Cappelletti , M. Barbieri , A. Aliprandi , M. Maggini , L. Dordevic , Nanoscale 2024, 16, 9153. 38639760 10.1039/d4nr00383g PMC11097008

[15] J. Su , Y. Song , Z. Zhu , X. Huang , J. Fan , J. Qiao , F. Mao , Signal Transduct. Target. Ther. 2024, 9, 196. 39107318 10.1038/s41392-024-01888-z PMC11382761

[16] C. Lennicke , H. M. Cocheme , Mol. Cell. 2021, 81, 3691. 34547234 10.1016/j.molcel.2021.08.018

[17] H. Sies , R. J. Mailloux , U. Jakob , Nat. Rev. Mol. Cell. Biol. 2024, 25, 701. 38689066 10.1038/s41580-024-00730-2 PMC11921270

[18] X. Sun , Y. Dong , Y. Liu , N. Song , F. Li , D. Yang , Sci. China Chem. 2021, 65, 31.

[19] N. Liu , H. Ma , M. Li , R. Qin , P. Li , FlexMat 2024, 1, 269.

[20] M. Berggren , E. D. Glowacki , D. T. Simon , E. Stavrinidou , K. Tybrandt , Chem. Rev. 2022, 122, 4826. 35050623 10.1021/acs.chemrev.1c00390 PMC8874920

[21] M. Hjort , A. H. Mousa , D. Bliman , M. A. Shameem , K. Hellman , A. S. Yadav , P. Ekstrom , F. Ek , R. Olsson , Nat. Commun. 2023, 14, 4453. 37488105 10.1038/s41467-023-40175-3 PMC10366153

[22] I. Palama , F. Di Maria , I. Viola , E. Fabiano , G. Gigli , C. Bettini , G. Barbarella , J. Am. Chem. Soc. 2011, 133, 17777. 21951102 10.1021/ja2065522

[23] G. Barbarella , F. Di Maria , Acc. Chem. Res. 2015, 48, 2230. 26234700 10.1021/acs.accounts.5b00241

[24] L. Aloisio , M. Moschetta , A. Boschi , A. G. Fleitas , M. Zangoli , I. Venturino , V. Vurro , A. Magni , R. Mazzaro , V. Morandi , A. Candini , C. D’Andrea , G. M. Paterno , M. Gazzano , G. Lanzani , F. Di Maria , Adv. Mater. 2023, 35, e2302756. 37364565 10.1002/adma.202302756

[25] I. Viola , I. E. Palama , A. M. Coluccia , M. Biasiucci , B. Dozza , E. Lucarelli , F. Di Maria , G. Barbarella , G. Gigli , Integr. Biol. 2013, 5, 1057. 10.1039/c3ib40064f 23806977

[26] I. E. Palama , F. Di Maria , S. D’Amone , G. Barbarella , G. Gigli , J. Mater. Chem. B 2015, 3, 151. 32261935 10.1039/c4tb01562b

[27] I. E. Palama , G. Maiorano , F. Di Maria , M. Zangoli , A. Candini , A. Zanelli , S. D’Amone , E. Fabiano , G. Gigli , G. Barbarella , ACS Omega 2022, 7, 12624. 35474798 10.1021/acsomega.1c06677 PMC9026133

[28] M. Moros , F. Di Maria , P. Dardano , G. Tommasini , H. Castillo‐Michel , A. Kovtun , M. Zangoli , M. Blasio , L. De Stefano , A. Tino , G. Barbarella , C. Tortiglione , iScience 2020, 23, 101022. 32283525 10.1016/j.isci.2020.101022 PMC7155203

[29] H. Fu , X. Gao , X. Zhang , L. Ling , Cryst. Growth Des. 2021, 22, 1476.

[30] Y. Tsarfati , S. Rosenne , H. Weissman , L. J. W. Shimon , D. Gur , B. A. Palmer , B. Rybtchinski , ACS Cent. Sci. 2018, 4, 1031. 30159400 10.1021/acscentsci.8b00289 PMC6107864

[31] M. Jehannin , A. Rao , H. Colfen , J. Am. Chem. Soc. 2019, 141, 10120. 31173682 10.1021/jacs.9b01883

[32] A. J. Cox , A. J. DeWeerd , J. Linden , Am. J. Phys. 2002, 70, 620.

[33] G. Mie , Annalen der Physik 2006, 330, 377.

[34] G. Barbarella , L. Favaretto , G. Sotgiu , L. Antolini , G. Gigli , R. Cingolani , A. Bongini , Chem. Mater. 2001, 13, 4112.

[35] M. Zangoli , R. Mazzaro , E. Lunedei , E. Fabiano , I. Manet , A. Candini , A. Kovtun , M. Goudjil , A. Zanelli , S. Rozen , M. Gazzano , M. Baroncini , F. Di Maria , ACS Nano 2025, 19, 2245. 39780446 10.1021/acsnano.4c11681

[36] F. Di Maria , M. Zangoli , I. E. Palamá , E. Fabiano , A. Zanelli , M. Monari , A. Perinot , M. Caironi , V. Maiorano , A. Maggiore , M. Pugliese , E. Salatelli , G. Gigli , I. Viola , G. Barbarella , Adv. Funct. Mater. 2016, 26, 6970.

[37] F. C. Spano , Acc. Chem. Res. 2010, 43, 429. 20014774 10.1021/ar900233v

[38] N. J. Hestand , F. C. Spano , Chem. Rev. 2018, 118, 7069. 29664617 10.1021/acs.chemrev.7b00581

[39] T. F. Heinz , In Carbon Nanotubes, Springer, Berlin, Heidelberg, 2007, Ch. Chapter 11, p. 353.

[40] S. J. Tang , P. H. Dannenberg , A. C. Liapis , N. Martino , Y. Zhuo , Y. F. Xiao , S. H. Yun , Light Sci. Appl. 2021, 10, 23. 33495436 10.1038/s41377-021-00466-0 PMC7835369

[41] D. Ohayon , S. Inal , Adv. Mater. 2020, 32, e2001439. 32691880 10.1002/adma.202001439

[42] C. Manzoni , G. Cerullo , J. Opt. 2016, 18, 103501.

[43] M. J. Frisch , G. W. Trucks , H. B. Schlegel , G. E. Scuseria , M. A. Robb , J. R. Cheeseman , G. Scalmani , V. Barone , G. A. Petersson , H. Nakatsuji , X. Li , M. Caricato , A. V. Marenich , J. Bloino , B. G. Janesko , R. Gomperts , B. Mennucci , H. P. Hratchian , J. V. Ortiz , A. F. Izmaylov , J. L. Sonnenberg , D. Williams , F. Ding , F. Lipparini , F. Egidi , J. Goings , B. Peng , A. Petrone , T. Henderson , D. Ranasinghe , V.G. Zakrzewski , et al., Gaussian 16, Revision B.01, Gaussian Inc., Wallingford, CT, USA 2016.

[44] Y. Zhao , D. G. Truhlar , Theor. Chem. Acc. 2007, 120, 215.

[45] Y. Zhao , D. G. Truhlar , Acc. Chem. Res. 2008, 41, 157. 18186612 10.1021/ar700111a

[46] R. Li , J. Zheng , D. G. Truhlar , Phys. Chem. Chem. Phys. 2010, 12, 12697. 20733991 10.1039/c0cp00549e

[47] F. Weigend , R. Ahlrichs , Phys. Chem. Chem. Phys. 2005, 7, 3297. 16240044 10.1039/b508541a

[48] J. Tomasi , B. Mennucci , R. Cammi , Chem. Rev. 2005, 105, 2999. 16092826 10.1021/cr9904009

[49] V. Barone , M. Cossi , J. Phys. Chem. A 1998, 102, 1995.

[50] C. Adamo , D. Jacquemin , Chem. Soc. Rev. 2013, 42, 845. 23117144 10.1039/c2cs35394f

[51] F. Neese , WIREs Comput. Mol. Sci. 2022, 12, e1606.

[52] F. Neese , A. Hansen , D. G. Liakos , J. Chem. Phys. 2009, 131, 064103. 19691374 10.1063/1.3173827

[53] C. Riplinger , F. Neese , J. Chem. Phys. 2013, 138, 034106. 23343267 10.1063/1.4773581

[54] A. Hellweg , C. Hättig , S. Höfener , W. Klopper , Theor. Chem. Acc. 2007, 117, 587.

[55] F. Weigend , Phys. Chem. Chem. Phys. 2006, 8, 1057. 16633586 10.1039/b515623h

[56] M. Garcia‐Rates , U. Becker , F. Neese , J. Comput. Chem. 2021, 42, 1959. 34347890 10.1002/jcc.26726

[57] R. Dennington , T. A. Keith , J. M. Millam , GaussView, Version 6, Semichem Inc., Shawnee Mission, KS, USA 2016.

[58] C. F. Macrae , I. Sovago , S. J. Cottrell , P. T. A. Galek , P. McCabe , E. Pidcock , M. Platings , G. P. Shields , J. S. Stevens , M. Towler , P. A. Wood , J. Appl. Crystallogr. 2020, 53, 226. 32047413 10.1107/S1600576719014092 PMC6998782

[59] R. L. Martin , J. Chem. Phys. 2003, 118, 4775.

