# Supplementary material for: From Molecules to Bioaggregates: Unraveling the Photoexcitation Dynamics of Intracellularly Self‐Assembled Thiophene‐Based Fibers
Source: Small Sci. 2025 Jul 28;5(10):2500241. doi: 10.1002/smsc.202500241 (PMC12499485; doi:10.1002/smsc.202500241)
Supplement: Supplementary file 1 — Supplementary Material [file SMSC-5-2500241-s001.pdf]

SUPPLEMENTARY INFORMATION

From molecules to bio-aggregates: unraveling the photoexcitation dynamics of intracellularly self-assembled thiophene-based fibers

Filippo Monti†, Ludovico Aloisio†, Nicol Spallacci†, Mattia Zangoli, Antonella Treglia, Ariel Garcìa Fleitas, Michele Guizzardi, Soraia Flammini, Matteo Moschetta, Giuseppe Maria Paternò, Francesca Di Maria, Guglielmo Lanzani**

I.	Optical microscopy	p.S2
II.	Absorption spectra	p. S3
III.	DFT calculations	p. S4-S11
IV.	Time Resolved Photoluminescence	p. S12-S14
V.	Transient Transmission	p. S15-S20
VI.	Minimum Inhibitory Concentration	p. S21

I. Optical microscopy

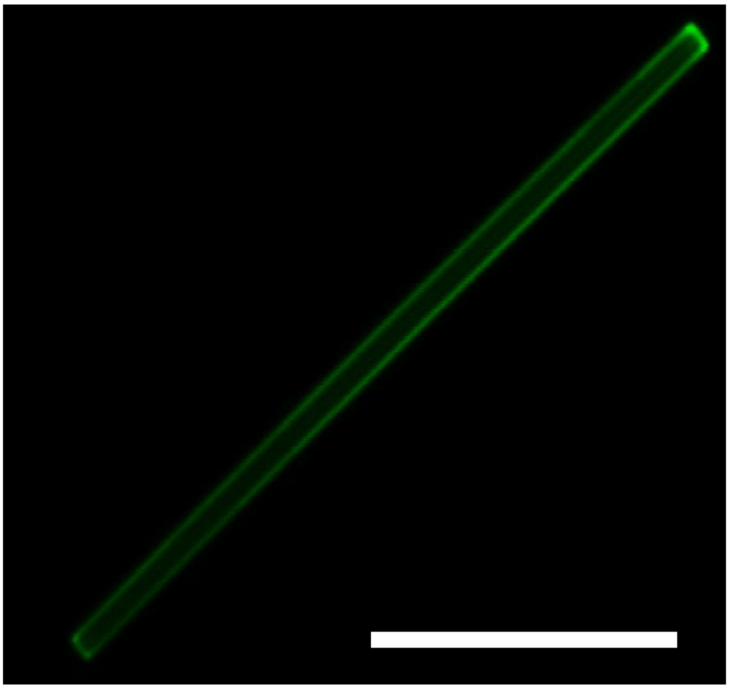

Figure S1: *Epifluorescent optical image of a DTTO crystal formed diluting the solution from DMSO into H_2O. Scale bar 25 μm.*

II. Absorption spectra

Scattering Baseline Subtraction

$$\mu_s' \approx a\left(\frac{\lambda}{\lambda_0}\right)^{-b} + c\left(\frac{\lambda}{\lambda_0}\right)^{-4} \qquad \text{(Equation S1)}$$

Where λ0 = 405 nm, a = 0.23397, b = 0.99365, c = 0.03069.

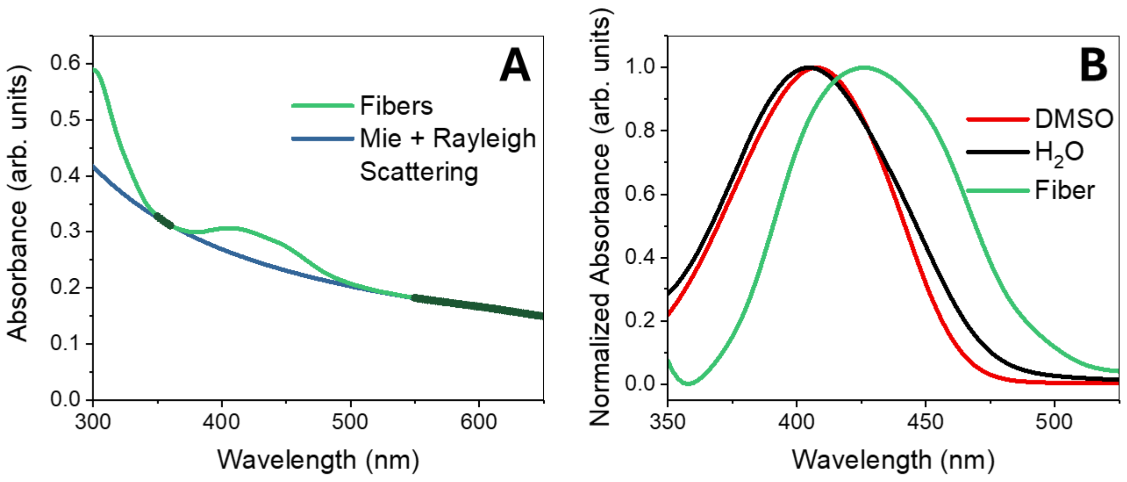

Figure S2: *(A) Absorption spectra collected on fibers' suspension in water (green line) and scattering baseline calculated considering both Mie and Rayleigh scattering (blue line). (B) Resulting absorbance spectrum of DTTO fibers suspended in water (green line), compared to DTTO in DMSO and in water.*

III. DFT calculations

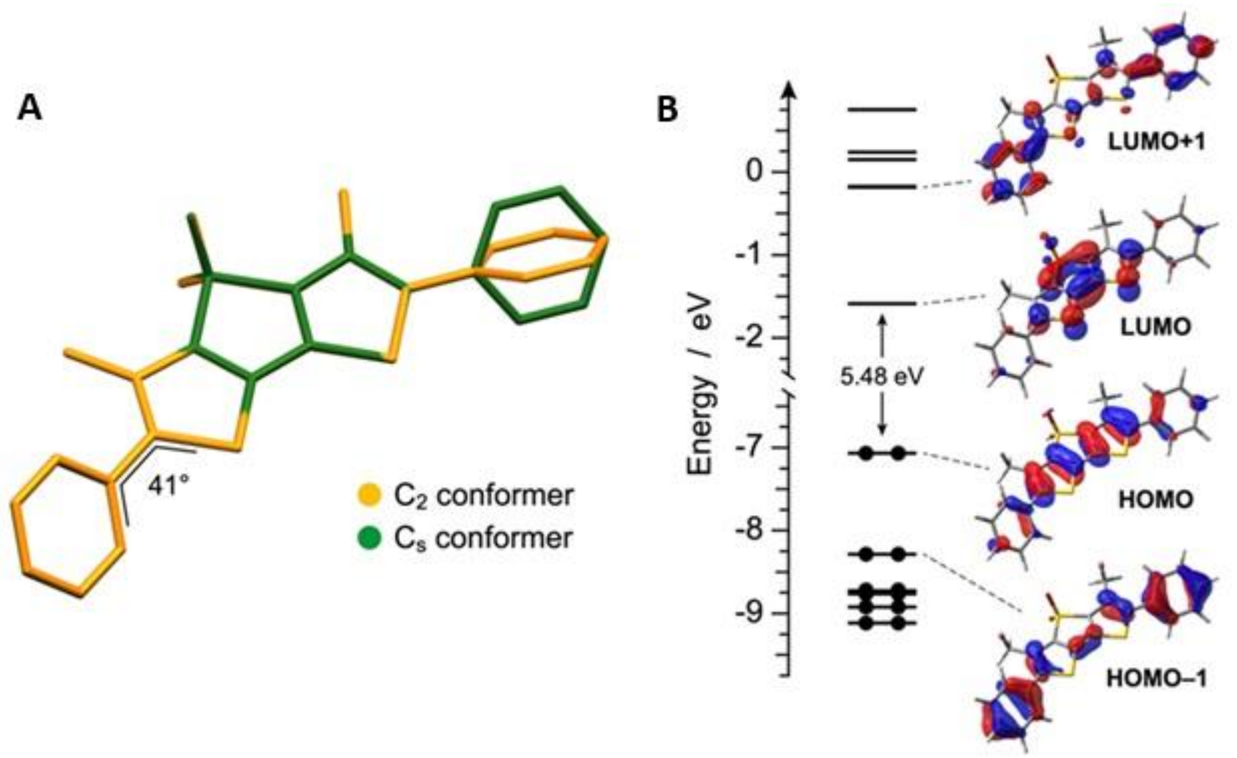

Figure S3. *A) Fully optimized ground-state geometries of the C_s and C_2 DTTO conformers. B) Energy diagram showing the energy values of the frontier Kohn-Sham molecular orbitals of DTTO (C_s symmetry); for relevant orbitals, the corresponding isosurfaces are also displayed (isovalue = 0.04 $e^{1/2}$ $bohr^{-3/2}$). All data are calculated at the M06-2X/def2-TZVP level of theory in DMSO (using PCM).*

Table S1. Calculated NTOs couples describing the lowest five singlet excitations (below 5.00 eV) for the C_2 DTTO conformer in DMSO at the PCM-TD-M06-2X/def2-TZVP level (see Experimental Section for details). The λ value is the natural transition orbital eigenvalue associated with each NTOs couple; orbital isovalue: 0.04 $e^{-1/2}$ $bohr^{-3/2}$. Transition symmetry is also reported, according to the C_2 point group.

	Transition energy [eV (nm)]	Oscillator strength	NTO couple hole	→ electron	(λ)
$S_0 \rightarrow S_1$ [B]	3.31 (374)	0.647	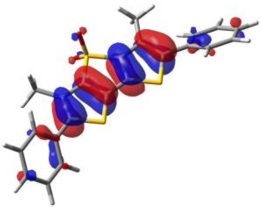	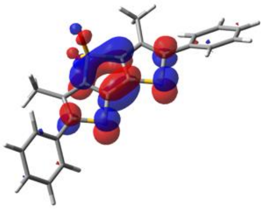	(98.2%)
$S_0 \rightarrow S_2$ [A]	4.73 (262)	0.066	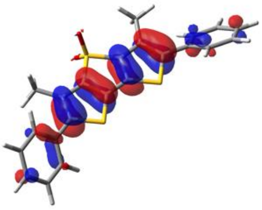	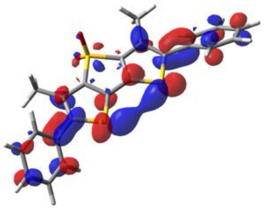	(70.0%)
$S_0 \rightarrow S_3$ [B]	4.78 (259)	0.169	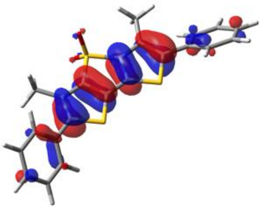	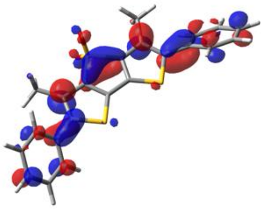	(55.2%)
$S_0 \rightarrow S_4$ [A]	4.86 (255)	0.028	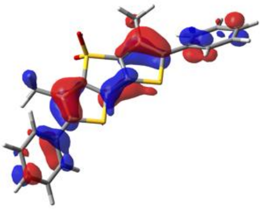	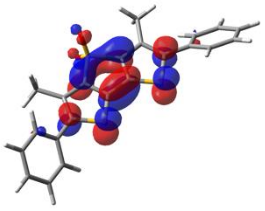	(79.0%)
$S_0 \rightarrow S_5$ [B]	4.90 (253)	0.637	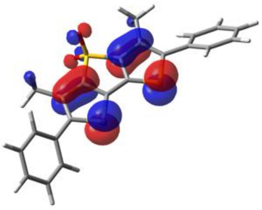	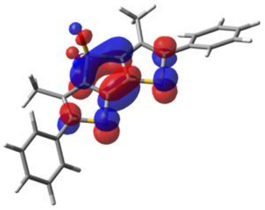	(61.0%)

Table S2. Calculated NTOs couples describing the lowest five singlet excitations (below 5.00 eV) for the C_s **DTTO** conformer in DMSO at the PCM-TD-M06-2X/def2-TZVP level (see Experimental Section for details). The λ value is the natural transition orbital eigenvalue associated with each NTOs couple; orbital isovalue: 0.04 $e^{-1/2}$ $bohr^{-3/2}$. Transition symmetry is also reported, according to the C_s point group.

	Transition energy [eV (nm)]	Oscillator strength	NTO couple<br>hole → electron<br>(λ)
$S_0 \rightarrow S_1$ [A"]	3.31 (374)	0.651	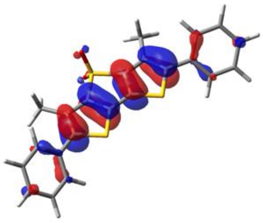 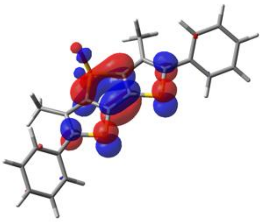 (98.1%)
$S_0 \rightarrow S_2$ [A"]	4.71 (263)	0.267	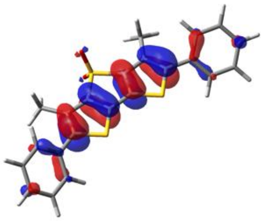 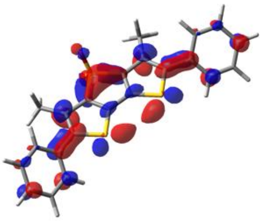 (83.1%)
$S_0 \rightarrow S_3$ [A']	4.80 (258)	0.087	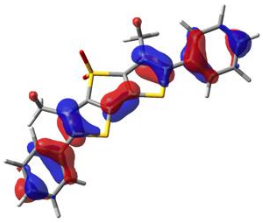 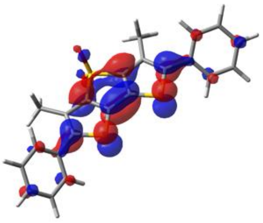 (70.3%)
$S_0 \rightarrow S_4$ [A"]	4.86 (255)	0.175	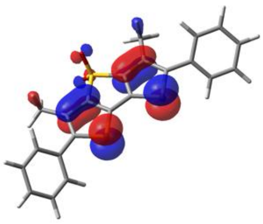 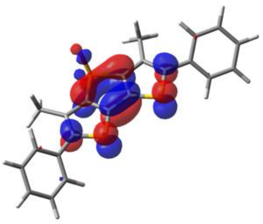 (84.3%)
$S_0 \rightarrow S_5$ [A']	4.92 (252)	0.005	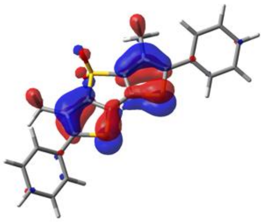 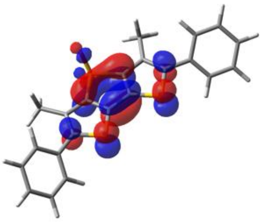 (54.1%)

Table S3. Calculated NTOs couples describing the lowest eight singlet excitations (below 5.00 eV) for **DTTO** in DMSO at the CPCM-STEOM-DLPNO-CCSD/def2-TZVP(-f) level, adopting the S_0 minimum-energy geometry in C_s symmetry, obtained by TD-DFT optimization (see Experimental Section for details). The λ value is the natural transition orbital eigenvalue associated with each NTOs couple; orbital isovalue: 0.04 $e^{-1/2}$ $bohr^{-3/2}$.

	Transition energy [eV (nm)]	Oscillator strength	NTO couple hole → electron (λ)
$S_0 \rightarrow S_1$	3.08 (403)	0.450	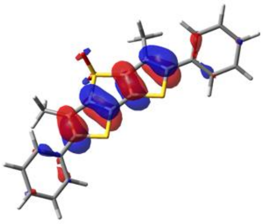 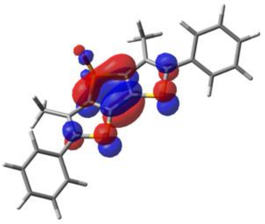 (96.3%)
$S_0 \rightarrow S_2$	4.33 (287)	0.064	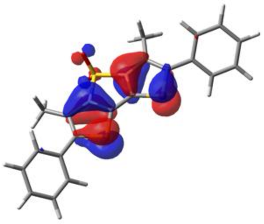 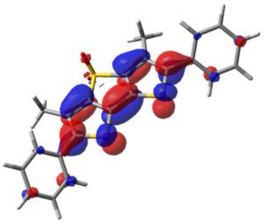 (51.6%)
$S_0 \rightarrow S_3$	4.49 (276)	0.003	Highly multiconfigurational *[No NTO couple with λ > 50%.]*
$S_0 \rightarrow S_4$	4.50 (275)	0.009	Highly multiconfigurational *[No NTO couple with λ > 50%.]*
$S_0 \rightarrow S_5$	4.52 (274)	0.001	Highly multiconfigurational *[No NTO couple with λ > 50%.]*
$S_0 \rightarrow S_6$	4.53 (273)	0.009	Highly multiconfigurational *[No NTO couple with λ > 50%.]*
$S_0 \rightarrow S_7$	4.71 (263)	0.293	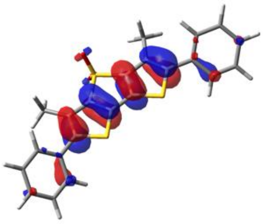 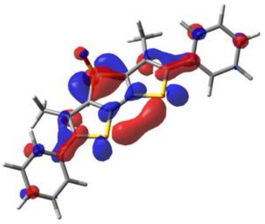 (53.6%)
$S_0 \rightarrow S_8$	4.95 (250)	0.454	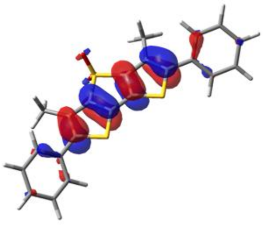 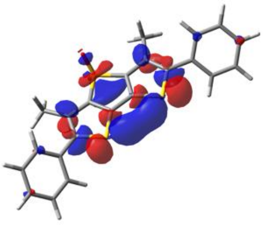 (76.4%)

Table S4. Transient absorption transitions (relative to S_1) of DTTO, in C_s symmetry, at the CPCM-STEOM-DLPNO-CCSD/def2-TZVP(-f) level in DMSO, adopting the S_1 minimum-energy geometry obtained by TD-DFT optimization (see Experimental Section for details).

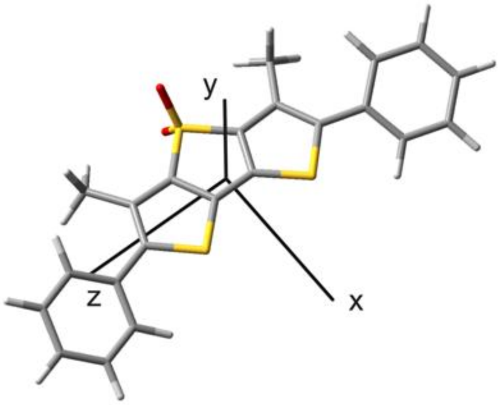

	Transition energy [eV (nm)]	Oscillator strength	Transition electric dipole moment [Debye]
$S_1 \rightarrow S_2$	1.54 (803)	0.054	[3.570, 0.211, -0.018]
$S_1 \rightarrow S_3$	1.66 (746)	1.297	[0.004, 0.000, -15.719]
$S_1 \rightarrow S_4$	1.88 (660)	0.000	[-0.191, 0.115, 0.022]
$S_1 \rightarrow S_5$	2.00 (622)	0.488	[0.142, 0.008, -8.279]
$S_1 \rightarrow S_6$	2.11 (587)	0.348	[0.001, 0.000, 6.993]
$S_1 \rightarrow S_7$	2.11 (587)	0.001	[-0.065, 0.225, 0.167]
$S_1 \rightarrow S_8$	2.22 (559)	0.011	[1.379, -0.346, -0.012]

Table S5. Calculated NTOs couples describing the lowest four singlet excitations for the H-type DTTO π-staked antiparallel dimer in DMSO (see Experimental Section for details). The λ value is the natural transition orbital eigenvalue associated with each NTOs couple; orbital isovalue: 0.04 $e^{-1/2}$ $bohr^{-3/2}$. Transition symmetry is also reported, according to the C_{2h} point group.

	Transition energy [eV (nm)]	Oscillator strength	NTO couple hole → electron (λ)
$S_0 \rightarrow S_1$ [B_g]	3.05 (407)	0.000	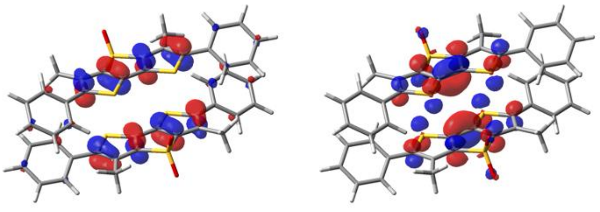 (83.9%)
$S_0 \rightarrow S_2$ [A_u]	3.29 (377)	1.164	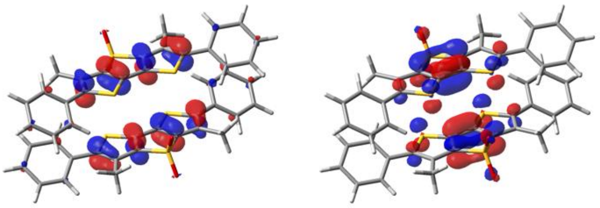 (52.9%)
$S_0 \rightarrow S_3$ [A_u]	3.85 (322)	0.008	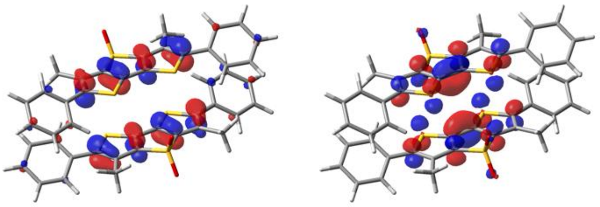 (53.5%)
$S_0 \rightarrow S_4$ [B_g]	3.98 (311)	0.000	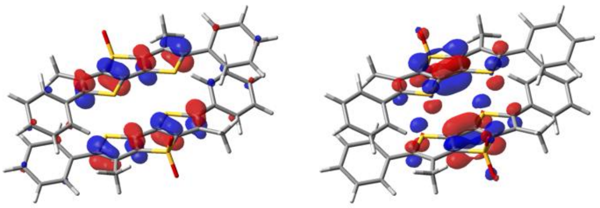 (83.6%)

Table S6. Calculated NTOs couples describing the lowest four singlet excitations for the M-type DTTO displaced π-staked antiparallel dimer in DMSO (see Experimental Section for details). The λ value is the natural transition orbital eigenvalue associated with each NTOs couple; orbital isovalue: 0.04 $e^{-1/2}$ $bohr^{-3/2}$. Transition symmetry is also reported, according to the C_i point group.

	Transition energy [eV (nm)]	Oscillator strength	NTO couple hole → electron (λ)
$S_0 \rightarrow S_1$ [A_u]	3.16 (392)	1.054	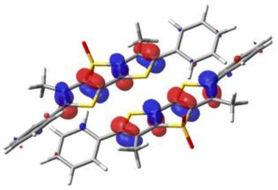 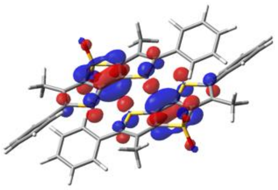 (81.1%)
$S_0 \rightarrow S_2$ [A_g]	3.17 (391)	0.000	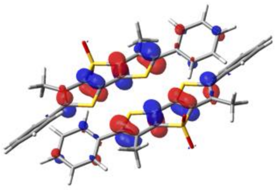 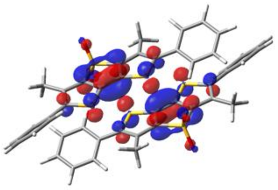 (49.1%)
$S_0 \rightarrow S_3$ [A_g]	3.78 (328)	0.000	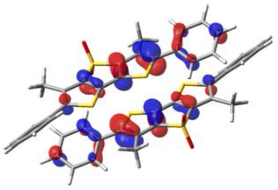 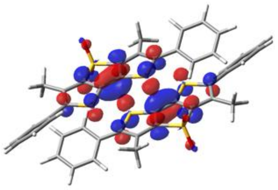 (51.0%)
$S_0 \rightarrow S_4$ [A_u]	3.88 (319)	0.187	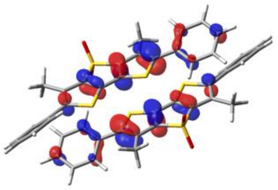 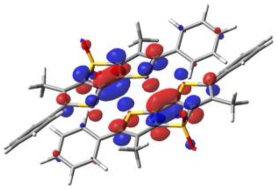 (82.2%)

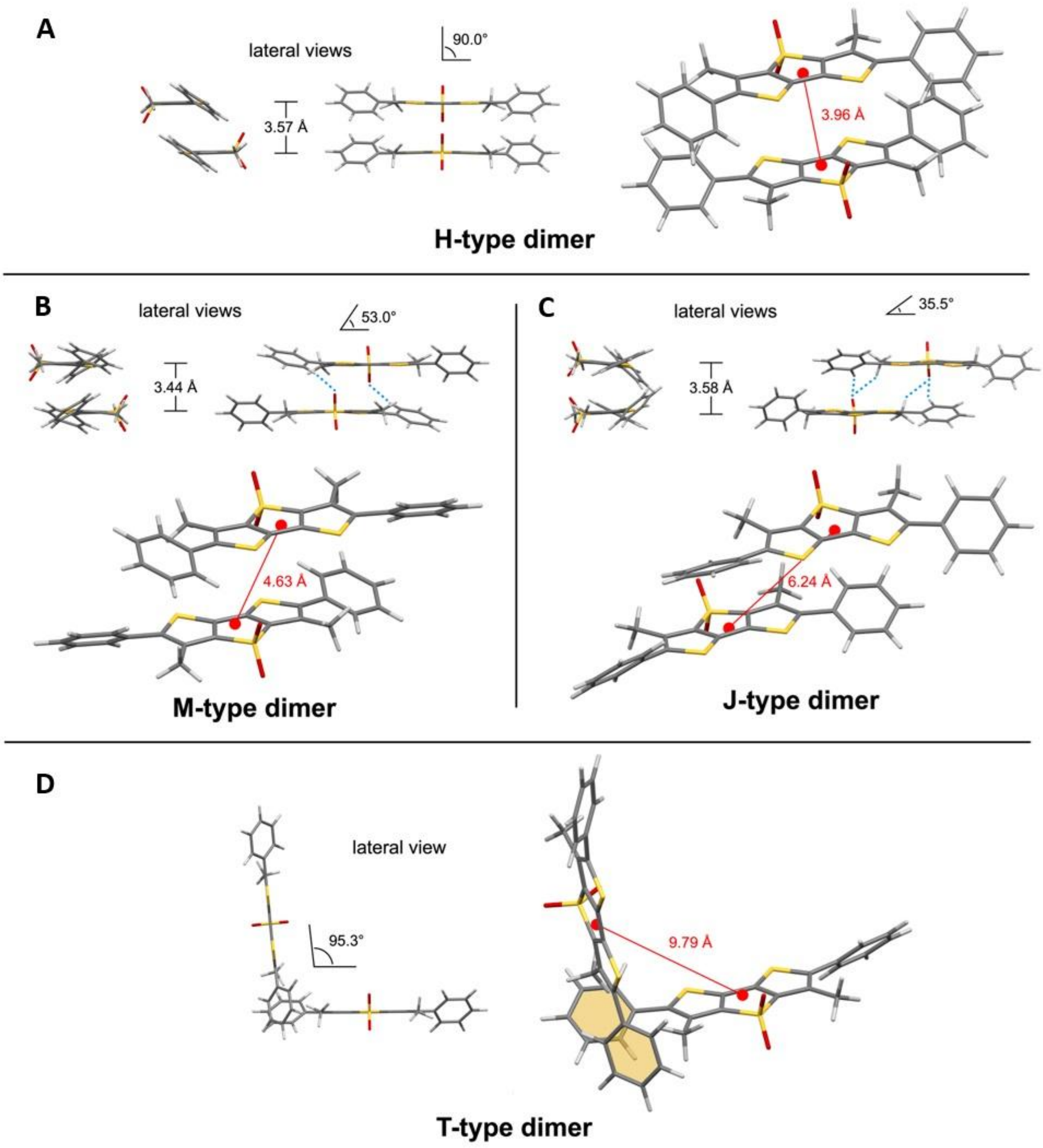

Figure S4. *Fully relaxed DTTO model dimers in DMSO. A) Absolute minimum (ΔE = – 0.72 eV vs. isolated monomers), obtained by maximizing the strong permanent dipole–dipole interaction between the single DTTO units; this dimer belongs to the C_{2h} point group, leading to a pure H-type antiparallel π–stacked dimer. B, C) Relative minima (ΔE = – 0.60 eV), displaying C–H···O=S intramolecular interactions between the peripheral DTTO substituents (phenyl and/or methyl groups) and the central SO_2 moiety (blue dotted lines); these dimers have a reduced symmetry: (B) belongs to the C_i point group, with the monomeric units still in an antiparallel π–stacked arrangement; (C) has C_2 symmetry and the DTTO molecules with aligned permanent dipoles (i.e., SO_2 units on the same side), leading to a wider interplanar distance (i.e., 3.58 vs. 3.44 Å). Dimer (B) is fundamentally a M-type aggregate (θ = 53.0°, very close to the magic angle θ_M = 54.7°), while (C) display an even smaller θ, belonging to the J-type category. D) T-type dimer with negligible intermolecular interactions between the peripheral DTTO substituents (ΔE = – 0.24 eV vs. isolated monomers).*

IV. Time Resolved Photoluminescence

Equation S2 describes the transition dipole moment (squared) of the bright state (optically allowed state) in a J-aggregate, which scales with the number of monomers in the 1D chain.

$$M_1^2 = \frac{2\mu^2}{N+1}\left(\frac{1}{tan\left(\frac{\pi}{2(N+1)}\right)}\right)^2 \qquad \text{(Equation S2)}$$

Where M represents the transition dipole moment of the optically allowed exciton state in the J-aggregate; μ is the transition dipole moment of a single monomer; N is the number of monomers in the linear (1D) J-aggregate chain.

Stoke's shift depends on the polarity and polarizability of the medium and, considering **Equation S3**, DTTO in DMSO has a Stoke shift of 615 meV (which corresponds well to experimental data), while this is larger for water, where it is predicted to be 750 meV.

To verify that this emission is coming from dispersed molecules, the same sample was prepared and let rest for 48 hours to favor the formation of crystalline aggregates. The sample appeared as large crystalline flakes suspended in a clear solution.

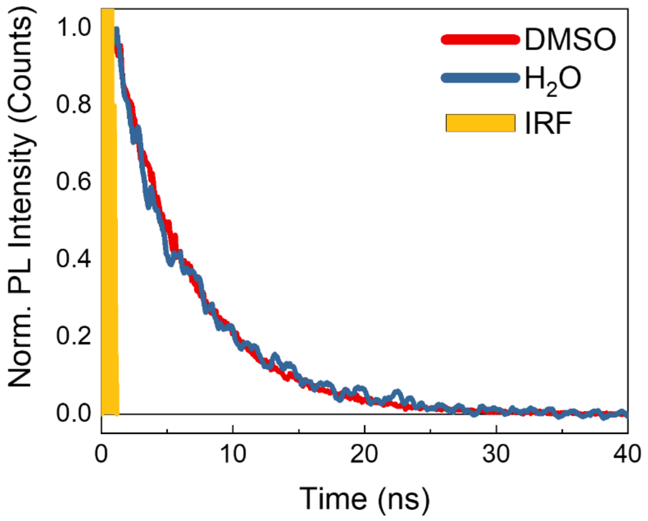

Figure S5: *Normalized PL intensity decay at long delays (from 1.5 ns onwards) of DTTO in DMSO and water.*

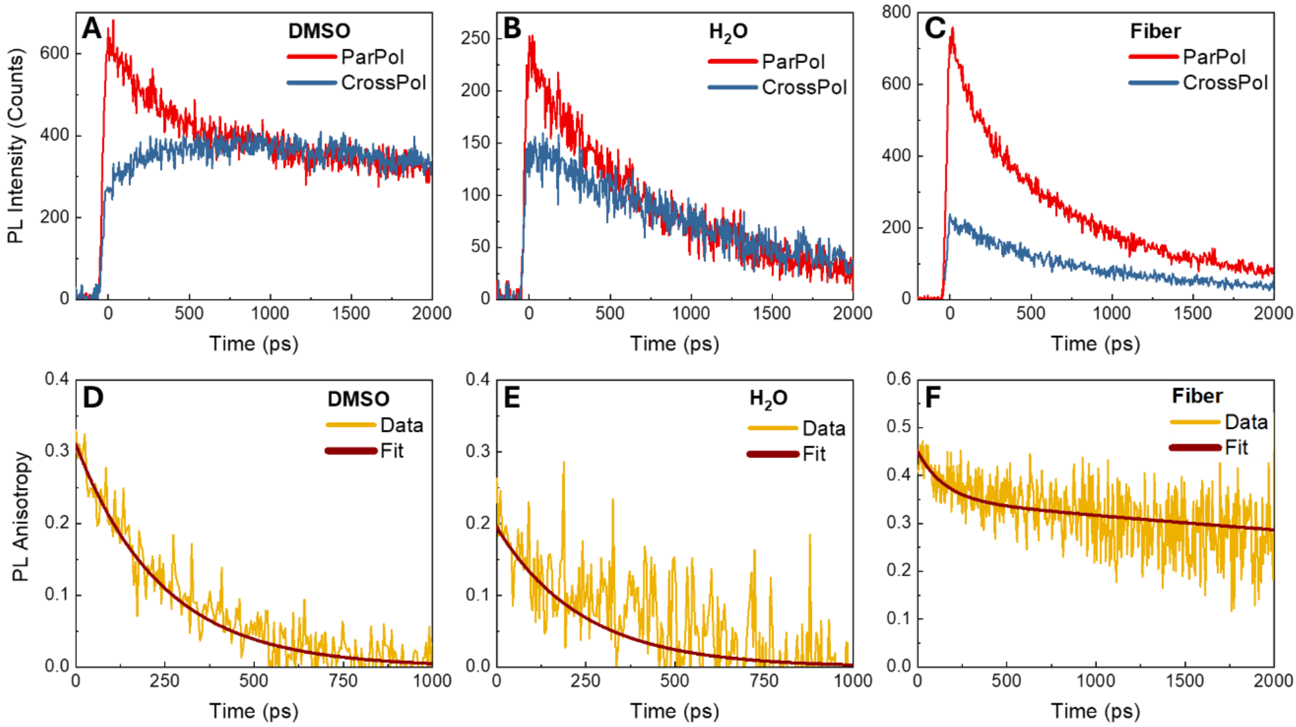

Figure S6: *PL intensity of DTTO in (A) DMSO, (B) water, (C) fibers vs time delay after excitation of emission polarized parallel (red line) or perpendicular (blue) to the excitation polarization, integrated over the whole spectrum. PL anisotropy decays of DTTO in (D) DMSO, (E) water, (F) fibers isolated from cell and deposited on a glass substrate to avoid signal from DTTO in cells.*

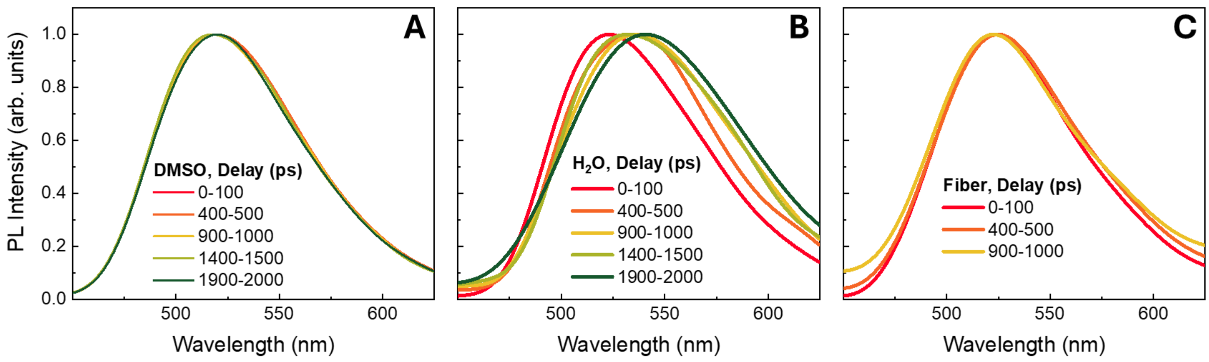

Figure S7: *Normalized PL spectra measured at different delays after excitation for DTTO in (A) DMSO, (B) water and (C) fibers. Spectra result from the integration of signal over 100 ps.*

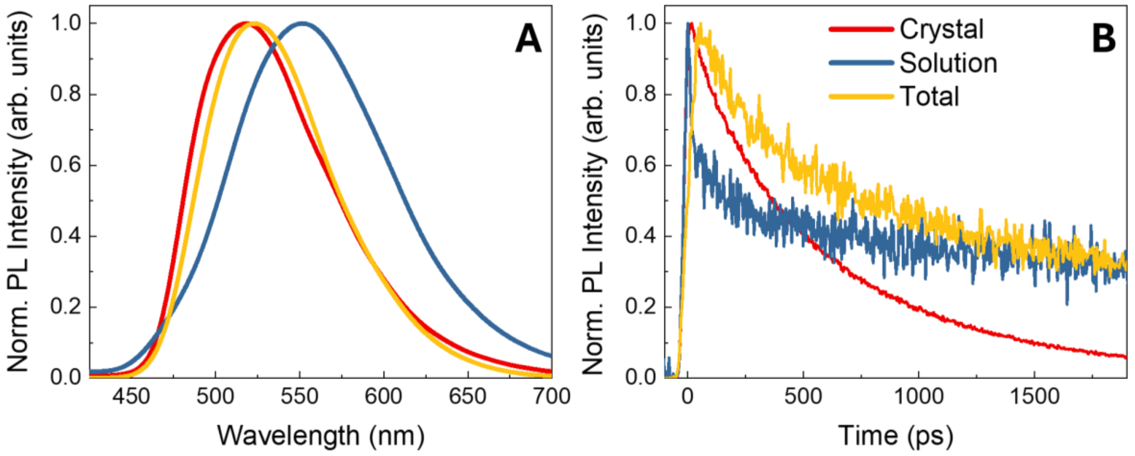

Figure S8: *(A) PL spectra integrated over the whole time delay (2000 ps) for DTTO aggregates formed in water. (B) Intensity decay time integrated over the whole spectra for the same samples.*

Figure S8 shows the data obtained measuring the crystals and the clear solution: the crystal behaves similarly to the fibers themselves, while the clear solution, where a very small fraction of molecules is present, shows a clearly red shifted spectrum, with a very fast initial decay, which can be ascribed to non-emitting H-aggregates and a longer component coming from the dispersed monomer. This confirms again that DTTO dispersed in water emits at lower energies with a long PL lifetime, as hypothesized in the main text.

$$\Delta E = \frac{|\Delta\mu|^2}{a^3}\left[\frac{\varepsilon - 1}{2\varepsilon + 1} - \frac{n^2 - 1}{2n^2 + 1}\right] \qquad \text{(Equation S3)}$$

V. Transient Transmission

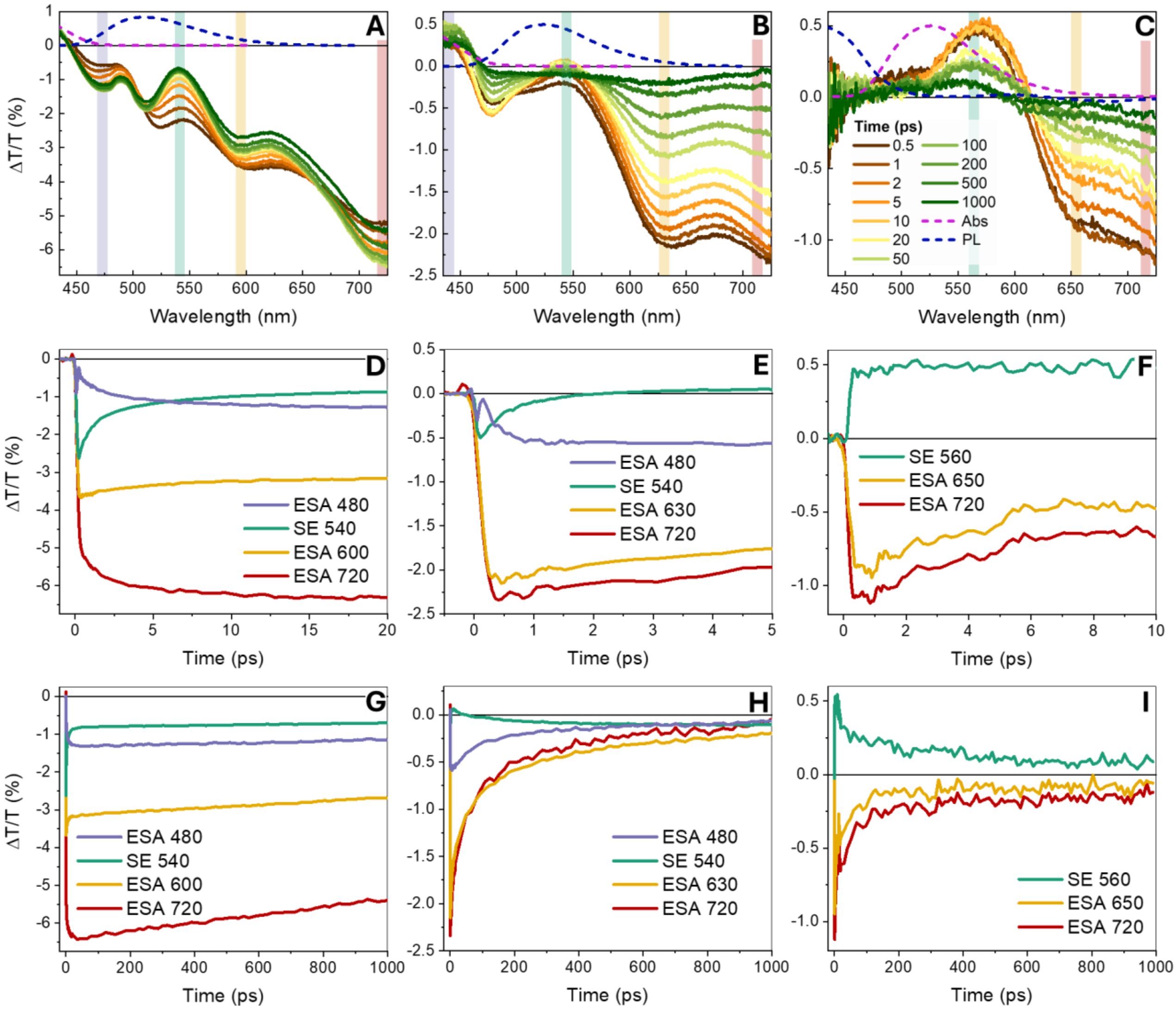

Figure S9: *Transient transmission spectra over time of DTTO in (A) DMSO, (B) water, (C) fibers. The time evolution is indicated by the color gradient of the line (from brown = 0.5 ps to green = 1000 ps). Dashed lines represent absorption and PL spectra measured in CW. (D-F) Normalized TA signal decay at specific wavelengths up to 20 ps after pump pulse, for DTTO in DMSO, water, and fibers, respectively. (G-I) Normalized TA signal decay at specific wavelengths up to 1 ns, for DTTO in DMSO, water, and fibers, respectively. Corresponding spectral areas of kinetics are highlighted in the spectra above.*

The model of the dielectric function of DTTO was built using classical oscillators, as represented in Equation 1, where A_k is the oscillator strength of the k transition, ω_k its angular frequency and γ_k is

$$\varepsilon_k = 1 + \sum_k \frac{A_k}{\omega_k^2 - \omega^2 - j\gamma_k \omega} \qquad \text{(Equation S4)}$$

the damping coefficient.

The model considers a few essential resonances: $k = 0$ for the ground state transition and $k = 1, 2, 3$ for the excited state transitions, and SE. The oscillator strength of each transition, $A_k(t)$, is time dependent and accounts for the generation and decay of the corresponding transition amplitude. This model is clearly oversimplified, because the only scope is to demonstrate the role of scattering. In the standard approach the ΔT/T signal under the well-separated pulses approximation, is accounted for by the pump-induced change in the imaginary part of the dielectric function,

$$\frac{\Delta T}{T}(\omega, t) = -\frac{\omega}{cn} L\{Im[\varepsilon_X(t)] - Im[\varepsilon_0]\} + SE(t) \qquad \text{(Equation S5)}$$

according to

c is the speed of light in vacuum, *n* the refractive index, *L* the sample thickness

Using this equation, we can reproduce well the ΔT/T spectra of the monomer in solution and water, retrieving quantitative values for the transition energies and their associated spectra and kinetics. To reproduce the spectra measured in fibers', we assume that the signal is predominantly due to scattered light, with a surviving transmission component only present well out of ground state resonance (ESA is less affected by scattering). The scattering cross section (Equation 3) in the ground (*G*) and excited (*X*) state depends on the complex dielectric function:

$$\frac{\Delta T}{T} = \frac{scatt_X - scatt_G}{scatt_G} - \frac{\omega}{cn} L\{Im[\varepsilon_X\,(t)]\} + SE \qquad \text{(Equation S6)}$$

The ΔT/T signal is thus given by:

The numerical simulation, in Figure 4I, and associated kinetics (SI) reproduces the experimental data in Figure 4C, supporting the hypothesis that scattering dominates the ΔT/T signal in the GSB spectral region.

$$N_{S_1} = \frac{1}{N_{S_1}(0)^{-1} + C_0 t} \qquad \text{(Equation S7)}$$

Equation S7 was used to account for bimolecular recombination, where the decay rate depends on the population of the excited state. This process, often observed in systems with interacting excitations, leads to a non-exponential decay.

The parameters derived from the MATLAB model (Tables S7, S8, and S9) are reported below.

Table S7: *Fitting values used in the MATLAB model for spectral prediction and dynamics of each transition involved in DTTO in DMSO. The reported amplitudes are relative amplitudes, intensities for each transition varies.*

**DMSO**	A_1	τ_1 (ps)	A_2	τ_2 (ps)	τ_{RAISE} (ps)
**GSB, SE, ESA 480, ESA 550, ESA 720**	0.94	5757	0.06	150	4

Table S8: *Fitting values used in the MATLAB model for spectral prediction and dynamics of each transition involved in DTTO aggregates suspended in water. The reported amplitudes are relative amplitudes, intensities for each transition varies.*

**Water**	$N_{S1}(0)$	C_0	A_1	τ_1 (ps)	A_2	τ_2 (ps)	τ_{RAISE} (ps)
**GSB, ESA 480, ESA 550, ESA 720**	0.54	0.046	0.33	350	0.013	5757	-
**SE**	0.54	0.046	0.33	350	0.013	5757	1
**CT**	-	-	1	2.11×10^9	-	-	20

Table S9: *Fitting values used in the MATLAB model for spectral prediction and dynamics of each transition involved in DTTO fibers suspended in water. The reported amplitudes are relative amplitudes, intensities for each transition varies.*

**Fibres**	$N_{S1}(0)$	C_0	A_1	τ_1 (ps)	A_2	τ_2 (ps)	τ_{RAISE} (ps)
**GSB**	-	-	-	-	-	-	-
**SE**	0.6	0.2	0.11	350	0.08	5757	4
**ESA 480**	0.58	0.19	0.12	350	0.09	5757	-
**ESA 590**	-	-	0.6	20	0.4	200	-
**ESA 720**	0.41	0.41	0.17	350	0.28	5757	-

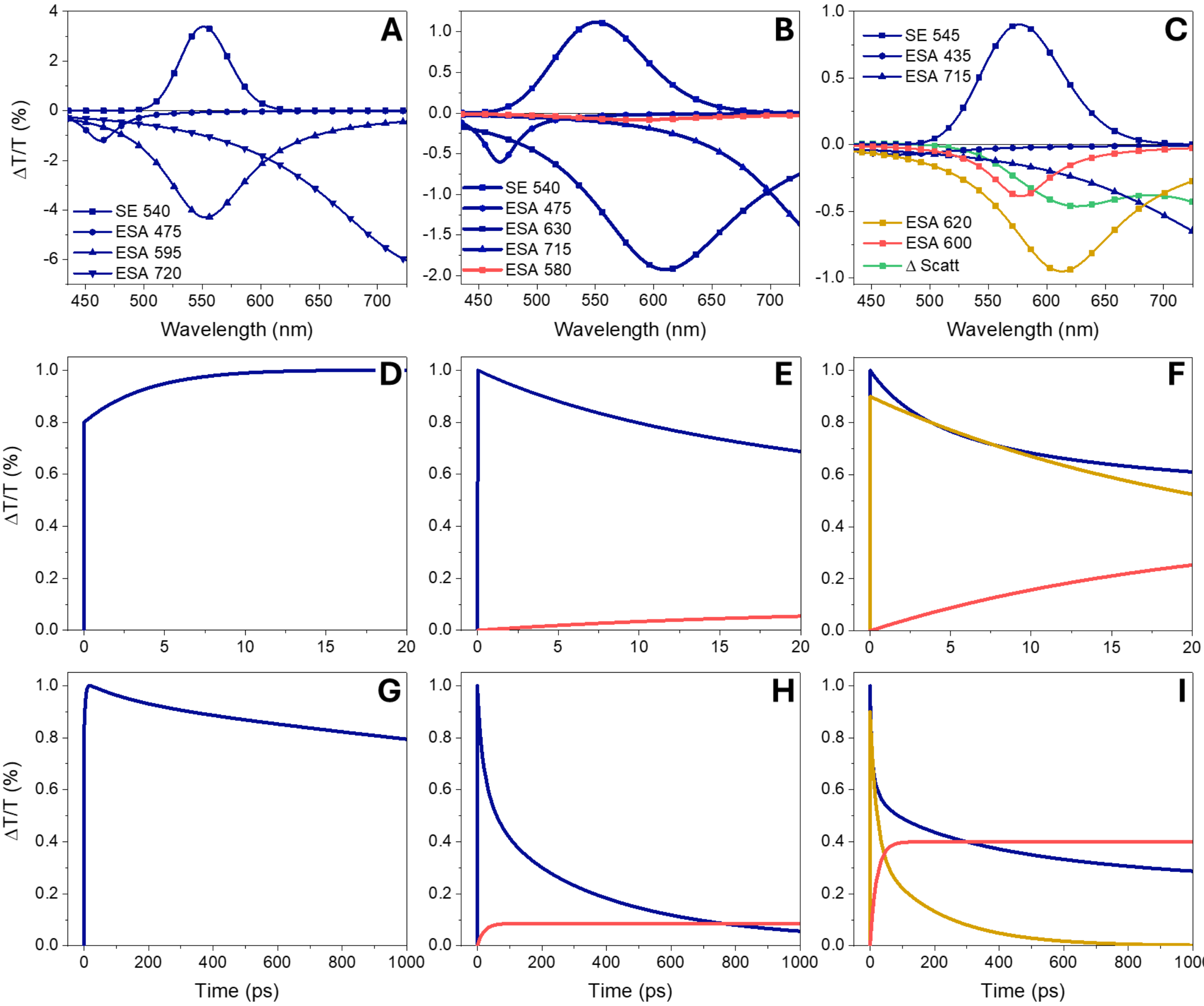

Figure S10: *Single component representing each transition composing the TA spectra of DTTO in (A) DMSO, (B) water and (C) fibers at 10, 5 and 5 ps delay respectively. Transitions of the same color are associated with the same time constants (τ) represented in the decays reported on the bottom: predicted pump probe signal decay for DTTO in (D, G) DMSO, (E, H) water, and (F, I). Dynamics are reported at two different timescales for clarity.*

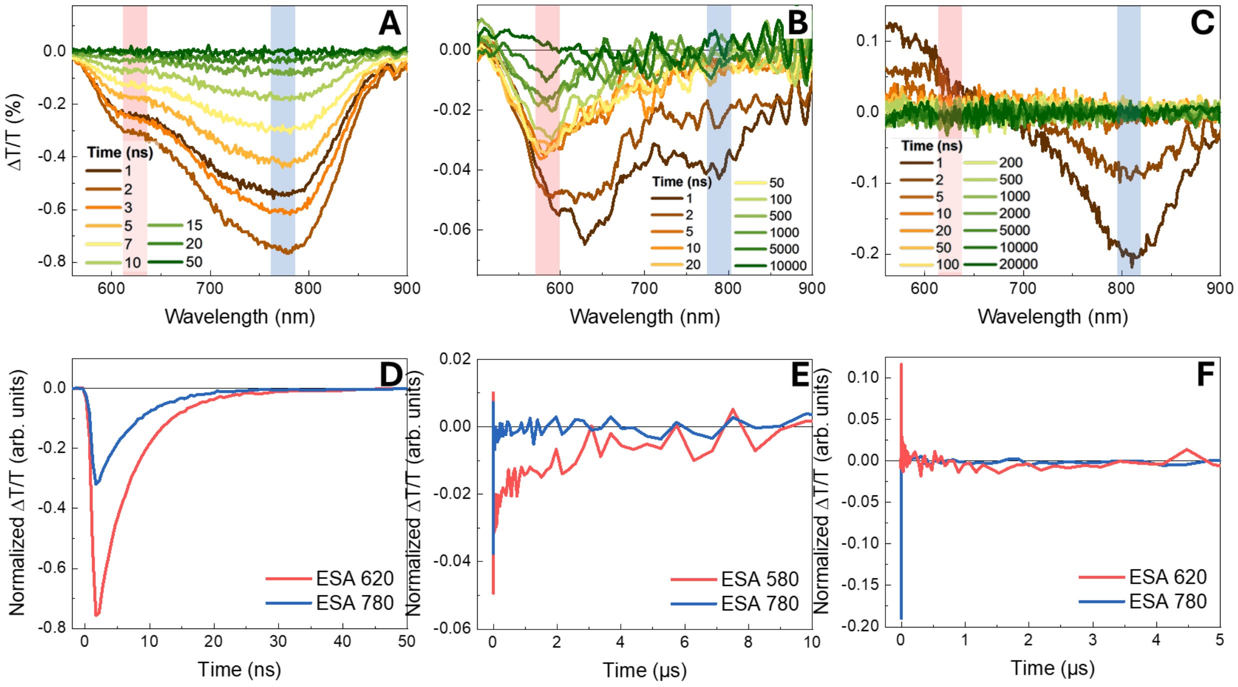

Figure S11: *Transient transmission spectra over time of (A) DTTO in DMSO, (B) DTTO in water, and (C) DTTO fibers at long delays. The time evolution is indicated by the color gradient of the line (from brown = 1 ps to green = 50 ns for DMSO, from brown = 1 ps to green = 20 µs for DTTO fibers). (D) Normalized TA signal decay at specific wavelengths up to 50 ns after pump pulse for DTTO in DMSO. (E) Normalized TA signal decay at specific wavelengths up to 10 µs after pump pulse for DTTO in water (F) Normalized TA signal decay at specific wavelengths up to 20 µs, for DTTO fibers. Corresponding spectral areas of kinetics are highlighted in the respective spectra.*

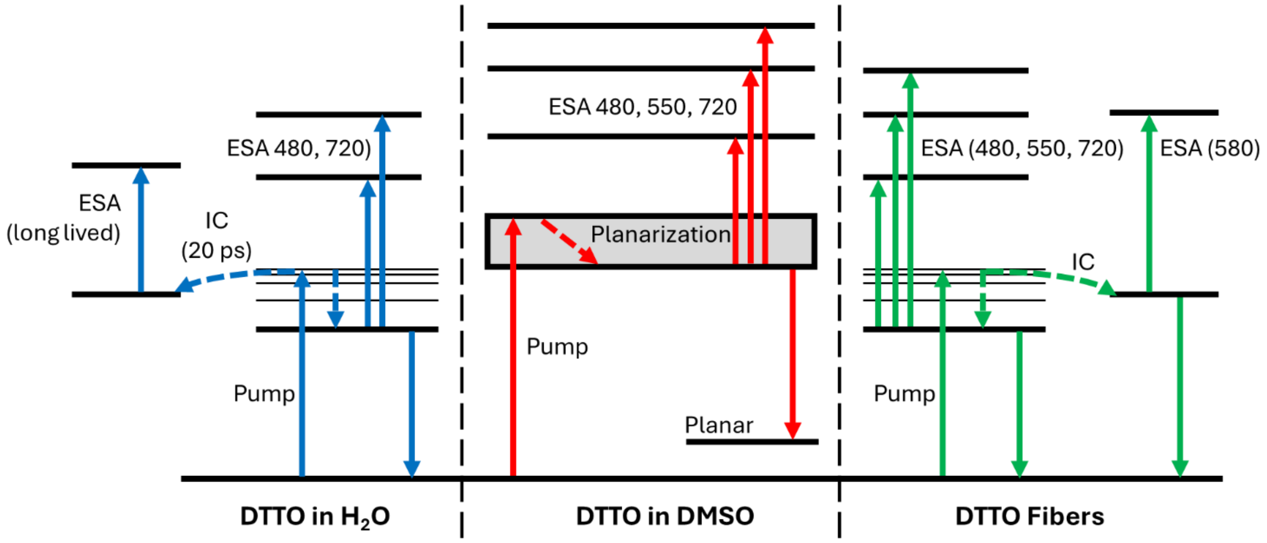

Figure S12: *Jablonski diagram representing energy levels of DTTO in DMSO, DTTO aggregates suspended in water, and DTTO fibers, as suggested by transient absorption data.*

VI. Minimum Inhibitory Concentration

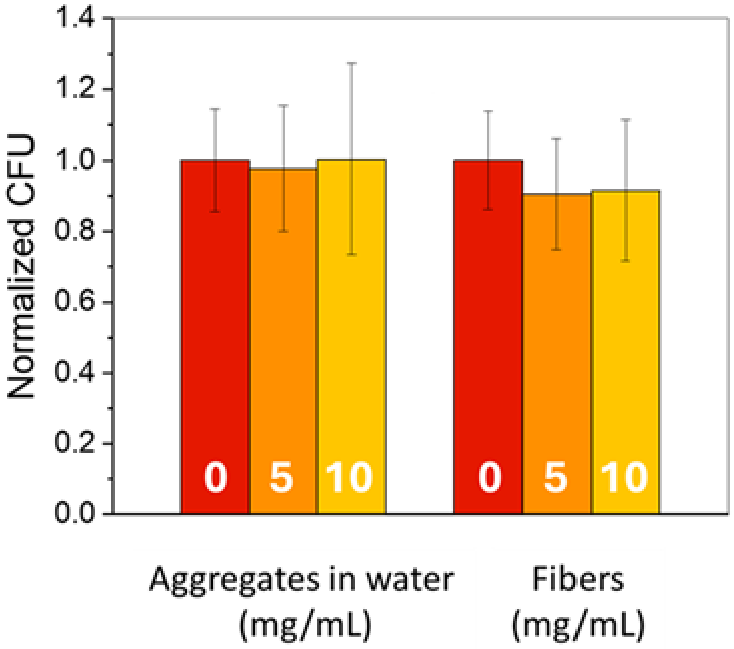

Figure S13: *Histogram representing the biocidal activity of DTTO aggregates formed in water (left) and DTTO fibers (right) against E. coli (0-10 µg/mL) under dark conditions. Data are expressed as mean ± SD.* H_2O*; 0µg/mL: 1±0.1440 (N=8), 5µg/mL: 0.9772±0.1760 (N=8), 10µg/mL: 1.003±0.2693 (N=7). Fibers; 0µg/mL: 1±0.1373 (N=10), 5µg/mL: 0.9044±0.1560 (N=8), 10µg/mL: 0.9154±0.1988 (N=10).*
